# A Triple-Hit Multi-Omics Framework for Psoriasis: Microbial Metabolic Remodeling and Immune Cell Methylome Signature Associated with an AMP-Dominant Lesional Program

**DOI:** 10.3390/life16030516

**Published:** 2026-03-20

**Authors:** Yoon Kyeong Lee, Hak Yong Kim, Donghwan Shim

**Affiliations:** 1 Department of Biochemistry, College of Natural Sciences, Chungbuk National University, Cheongju 28644, Republic of Korea; hykim@cbnu.ac.kr; 2 Department of Advanced Bio-Convergence, Chungbuk Health & Science University, Cheongju 28150, Republic of Korea; 3 Department of Biological Science, Chungnam National University, Daejeon 34134, Republic of Korea

**Keywords:** psoriasis, gut–skin axis, multi-omics, microbial metabolism, lipid metabolism, DNA methylation, immune cell epigenetics, antimicrobial peptides

## Abstract

The gut–skin axis is increasingly implicated in psoriasis pathogenesis, yet the cross-compartment convergence of molecular programs remains incompletely defined. We constructed a conceptual “Triple-Hit” multi-omics framework by integrating five independent public datasets spanning gut microbial functional remodeling (shotgun metagenomics), systemic immune cell methylomes (PBMC and CD8+ T-cell EPIC 850K), and lesional skin regulatory layers (miRNA and bulk RNA-seq). In the gut compartment, functional profiles exhibited a selective reduction in microbial lipid catabolic potential, including decreased fatty acid degradation and a lowered composite lipid degradation score, alongside heterogeneous shifts across SCFA-associated metabolic pathways. Systemically, PBMC methylomes revealed widespread regional remodeling (45,396 DMRs) enriched for membrane-proximal signaling and cytoskeletal programs, while CD8+ T cells showed specific epigenetic alterations in lipid- and glycosphingolipid-associated loci, suggesting a systemic metabolic–epigenetic alignment. In the skin, we identified a compact miRNA signature (168 DE-miRNAs) and a mechanistically interpretable, directionality-constrained miRNA–mRNA bridge that aligns with an AMP-dominant inflammatory transcriptome, consistent with reduced post-transcriptional restraint. Collectively, these findings support a convergent multi-omics framework linking putative microbial metabolic remodeling, systemic immune priming, and cutaneous effector programs. This study provides a systems-level perspective on psoriasis pathogenesis, highlighting the metabolic–epigenetic–transcriptional convergence as a potential avenue for therapeutic intervention.

## 1. Introduction

Psoriasis is a chronic, immune-mediated inflammatory disease traditionally defined by hyperproliferative skin lesions, but it is now widely recognized as a systemic disorder with metabolic, immunological, and microbial dimensions [1,2,3,4]. Beyond the visible cutaneous manifestations, patients frequently exhibit systemic inflammation and an increased prevalence of cardiometabolic comorbidities, highlighting the need for integrative models that extend beyond skin-restricted pathology [5,6]. In this context, the gut–skin axis has emerged as a conceptual framework linking intestinal microbial ecology, host immune regulation, and cutaneous inflammation, yet the molecular convergence across these compartments remains incompletely defined.

Accumulating evidence implicates gut microbial dysbiosis in psoriasis, with multiple studies reporting altered community composition and reduced diversity compared with healthy controls [7,8,9]. However, taxonomic descriptions alone offer limited mechanistic insight. Recently, the attention has shifted toward functional profiling, emphasizing microbial metabolic potential as a biologically interpretable layer connecting microbes to host physiology [10]. Importantly, such metagenome-derived profiles represent community-level functional capacity rather than direct measurements of metabolite concentrations. Short-chain fatty acid (SCFA)-associated pathways and lipid metabolism have been repeatedly implicated in inflammatory skin diseases. While SCFA depletion is often cited, recent findings suggest a broader remodeling of microbial lipid catabolic capacity, raising the possibility that higher-order metabolic shifts—rather than transient fluctuations of individual metabolites—may serve as persistent upstream signals in psoriasis pathophysiology [11,12,13].

In parallel, systemic epigenetic alterations in immune cells have been increasingly reported in psoriasis. Epigenome-wide association studies (EWASs) in peripheral blood mononuclear cells (PBMCs) and purified immune subsets have identified disease-associated DNA methylation changes at loci involved in immune identity, signaling, and cellular activation [14,15,16]. Crucially, microbial metabolites such as SCFAs have been reported to act as epigenetic modifiers (e.g., via histone deacetylase [HDAC] inhibition), suggesting a potential mechanistic link between gut metabolic remodeling and systemic immune states [17,18,19]. A key unresolved issue is whether such epigenetic changes represent “priming” mechanisms that predispose immune responses or merely secondary adaptations. Distinguishing between these interpretations requires integrative analyses that relate immune cell methylomes to upstream microbial functional signals and downstream tissue-level programs.

At the tissue level, psoriatic lesions are characterized by a robust inflammatory–proliferative transcriptional state driven largely by keratinocytes and infiltrating immune cells [20,21]. Antimicrobial peptides (AMPs) and alarmins, including members of the S100 family and β-defensins, are consistently upregulated and contribute to both barrier dysfunction and immune activation [22,23,24,25]. In addition to transcriptional control, post-transcriptional regulation by microRNAs (miRNAs) has emerged as an important layer shaping keratinocyte behavior, immune signaling, and barrier-related processes in psoriasis [26,27]. Nevertheless, how miRNA-mediated regulation aligns with systemic epigenetic signatures and microbial metabolic changes has not been systematically addressed within a unified framework.

Despite substantial progress across individual layers, several gaps remain. Most studies examine the gut microbiome, immune epigenome, or lesional transcriptome in isolation, often using distinct cohorts and platforms, limiting cross-compartment inference. Moreover, microbial metabolic remodeling, immune cell methylome signatures, and lesional effector programs are rarely evaluated within a coherent analytical framework that prioritizes biological alignment across layers. As a result, it remains unclear whether these features represent independent disease correlates or components of a convergent, system-level psoriasis-associated program.

Here, we present a conceptual “Triple-Hit” multi-omics framework that integrates gut microbial functional profiles, systemic immune cell DNA methylation signatures, and lesional regulatory and transcriptomic programs using five independent public datasets. By focusing on (i) putative gut trigger (microbial metabolic remodeling), (ii) putative systemic mediator (immune cell epigenetic priming), and (iii) downstream skin effector (AMP-dominant lesional state), we aim to identify cross-compartment patterns that are consistently associated with psoriasis. This integrative approach provides a structured framework for understanding how microbial, epigenetic, and cutaneous layers functionally align in psoriasis, highlighting systemic metabolic–epigenetic convergence, particularly in lipid- and membrane-proximal programs, as a key feature of disease pathogenesis. Because these datasets originate from independent cohorts, our cross-layer interpretations emphasize convergent associations rather than direct causal inference.

## 2. Materials and Methods

### 2.1. Study Design and Multi-Omics Dataset Integration

#### 2.1.1. The “Triple-Hit” Framework Design

**Figure 1:**
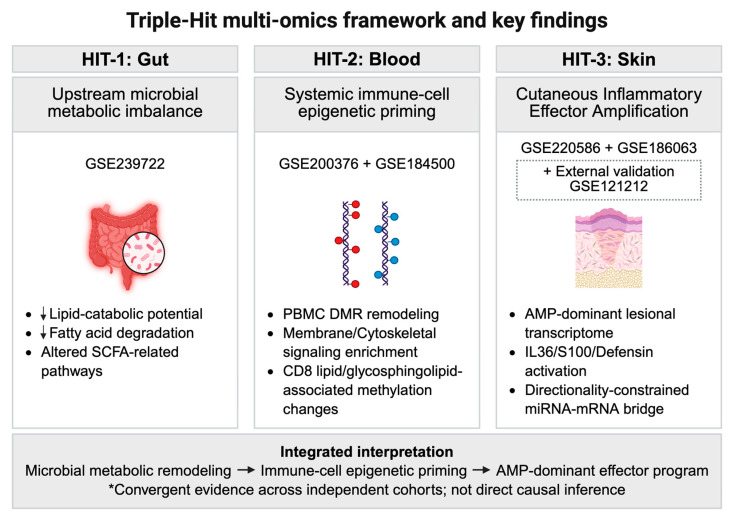
Triple-Hit multi-omics framework and key findings across the gut–blood–skin axis in psoriasis: The schematic summarizes the design logic and principal findings of the study across three biological compartments using independent public datasets. (**Hit 1: Gut**) represents upstream microbial metabolic imbalance identified by shotgun metagenomic profiling in GSE239722 (HC vs. PsO-UT), including reduced lipid catabolic potential, reduced fatty acid degradation, and altered short-chain fatty acid (SCFA)-related pathways. Downward arrows in the Hit 1 panel indicate decreases in the indicated features. (**Hit 2: Blood**) represents systemic immune cell epigenetic priming captured by EPIC 850K DNA methylation analyses in PBMCs (GSE200376; HC vs. PsO-PB) and purified CD8+ T cells (GSE184500; HC vs. PsO-CD8), highlighting PBMC DMR remodeling, membrane/cytoskeletal signaling enrichment, and CD8 lipid/glycosphingolipid-associated methylation changes. (**Hit 3: Skin**) represents cutaneous inflammatory effector amplification, integrating lesional miRNA profiling (GSE220586; HC vs. PsO-L) and lesional bulk RNA-seq (GSE186063; AS-HC vs. PsO-L), as well as highlighting an AMP-dominant lesional transcriptome, IL36/S100/defensin activation, and a directionality-constrained miRNA–mRNA bridge. Because the transcriptomic comparator in GSE186063 consisted of healthy-appearing skin from patients with ankylosing spondylitis, the major skin-level findings were further examined in an external healthy control validation cohort (GSE121212; bona fide HC vs. PsO-L), in which the principal inflammatory and AMP-associated lesional signals were reproduced. The integrated interpretation supports a convergent model linking microbial metabolic remodeling, immune cell epigenetic priming, and an AMP-dominant lesional skin effector program across independent cohorts, while not implying direct causal inference. The asterisk is intended to indicate an interpretive caveat, not a dataset-specific note. It means that the integrated framework is supported by convergent findings across independent cohorts and omics layers, but should not be interpreted as evidence of a direct causal relationship.

To reconstruct the systemic pathology of psoriasis along the gut–blood–skin axis, we designed a conceptual “Triple-Hit” multi-omics framework (Figure 1). This study integrates five independent public datasets spanning three biological compartments to investigate the following hypothesized layers:

1. Hit 1 (Putative Gut Trigger): microbial functional remodeling and metabolic potential.
2. Hit 2 (Putative Systemic Mediator): epigenetic priming in circulating immune cells (PBMCs and CD8+ T cells).
3. Hit 3 (Downstream Skin Effector): post-transcriptional regulation and inflammatory transcriptomic programs in lesional skin.

This design prioritizes cross-layer interpretability, aiming to connect microbial metabolic shifts to systemic epigenetic remodeling and cutaneous inflammation within a unified mechanistic hypothesis while acknowledging the cohort heterogeneity inherent to public data integration.

#### 2.1.2. Data Acquisition and Cohort Definitions

All datasets were retrieved from the NCBI Gene Expression Omnibus (GEO) or Sequence Read Archive (SRA). Cohort definitions and group assignments for each omics layer are summarized in Table 1 and Table S1.

• **Gut Microbiome (Metabolic Layer)**: Shotgun metagenomic sequencing data were obtained from GSE239722 [28]. To capture the baseline functional state without the confounding effects of systemic therapy, we selected the samples from untreated psoriasis patients (PsO-UT, *n* = 8) and healthy controls (HCs, *n* = 8).
• **Systemic Epigenome (Epigenetic Layer)**: To profile systemic epigenetic alterations, we utilized two independent DNA methylation datasets generated on the Illumina Infinium MethylationEPIC BeadChip (850K) platform. For the aggregate immune state, we compared peripheral blood mononuclear cells (PBMCs) from psoriasis vulgaris patients (PsO-PB, *n* = 20) with healthy controls (HCs, *n* = 19; GSE200376), while cell type-specific epigenetic priming was investigated using purified CD8+ T cells from psoriasis patients (PsO-CD8, *n* = 10) and healthy controls (HCs, *n* = 9; GSE184500) [29,30].
• **Skin Transcriptome and Regulome (Effector Layer)**: To construct the regulatory bridge in psoriatic lesions, we integrated lesional miRNA profiles (GSE220586; PsO-L *n* = 4 vs. HCs, *n* = 4) with lesional transcriptomic data (GSE186063; PsO-L, *n* = 13) [31,32]. For the transcriptomic comparator group in GSE186063, we used healthy-appearing skin from patients with ankylosing spondylitis (AS-HC, *n* = 12) as the available within-dataset baseline. These samples were not treated as bona fide disease-free skin controls; accordingly, GSE186063 was interpreted strictly as a within-dataset contrast. To assess the robustness of the major lesional transcriptomic findings against bona fide healthy skin controls, an independent external psoriasis skin RNA-seq cohort (GSE121212) was analyzed separately as a sensitivity/validation dataset [33,34].

#### 2.1.3. Dataset Selection Rationale and Cross-Cohort Bias Control

The datasets used in this study were not intended to represent an exhaustive survey of all publicly available psoriasis omics resources but rather as a design-driven set chosen to maximize biological interpretability across the gut–blood–skin axis. The dataset selection was guided by five prespecified principles: (i) compartment coverage across the putative gut trigger, systemic immune mediator, and downstream skin effector layers; (ii) public reanalyzability, including the availability of raw or sufficiently processable data; (iii) platform suitability for pathway-level interpretation within each layer, including shotgun metagenomics, EPIC 850K DNA methylation, skin miRNA profiling, and bulk RNA-seq; (iv) clinical interpretability, favoring cohorts with clearly defined healthy control and psoriasis-related groups and, where available, biologically informative features such as untreated status, purified immune cell fractions, or lesional skin phenotypes; and (v) complementarity rather than redundancy, such that each dataset contributed a distinct layer of information to the Triple-Hit framework rather than duplicating the same biological compartment.

Because the selected datasets originated from independent public cohorts, we did not perform direct sample-level pooling or batch-corrected cross-platform integration. Instead, each dataset was analyzed independently within its own cohort using within-dataset contrasts, and cross-layer interpretation was restricted to directionally concordant pathway-, regulator-, and phenotype-level signals. This strategy was adopted to reduce false comparability across platforms and study populations while preserving mechanistic interpretability. Preprocessing was defined according to data modality and analytical input level rather than imposed uniformly across all datasets: Noob normalization was applied to raw Illumina EPIC methylation datasets (GSE200376 and GSE184500), and TMM normalization was applied to count-level bulk RNA-seq datasets (GSE186063 and GSE121212). By contrast, GSE239722 was analyzed using author-provided processed microbial functional abundance profiles, and GSE220586 was analyzed using study-provided normalized miRNA expression values with current-study quality filtering; Noob/TMM was not re-applied to these datasets, because such procedures were not methodologically appropriate and could introduce redundant normalization. Additional bias control considerations included restricting the gut metagenomic analysis to untreated psoriasis samples in GSE239722, using purified immune cell methylomes to complement PBMC-level aggregate signals, and interpreting the GSE186063 skin transcriptomic comparison strictly as a within-dataset contrast with external healthy control validation provided separately by GSE121212. Age- and sex-adjusted modeling was applied in the PBMC methylation analysis (GSE200376), whereas a uniform age-adjusted framework could not be implemented across all datasets because age metadata were unavailable for GSE239722, GSE220586, and GSE121212.

GSE186063 was selected primarily because it provided a publicly reanalyzable lesional skin bulk RNA-seq resource that could be aligned with the skin miRNA layer within the effector component of the Triple-Hit framework. Importantly, this dataset was not selected because its comparator represented an ideal disease-free skin control. Rather, AS-HC was treated as an available within-dataset comparator that provided a conservative inflammatory background within the broader spondyloarthritis spectrum. Thus, lesional programs that remained prominent against this comparator were interpreted not as definitively psoriasis-specific, but as psoriasis-enriched signals that were less likely to reflect nonspecific inflammatory activation alone. Accordingly, major conclusions derived from GSE186063 were further tested in an independent psoriasis skin RNA-seq cohort with bona fide healthy controls (GSE121212).

The purified CD8+ T-cell methylome dataset was selected to represent a more specific immune cell epigenetic layer complementary to the PBMC-level methylation signals. This choice was hypothesis-driven: the present study aimed to capture an immune cell compartment plausibly linked to cytotoxic effector function, tissue residency, and downstream lesional inflammatory recall, all of which are biologically relevant to psoriasis persistence and recurrence. CD8+ T cells therefore provided a coherent cell type for evaluating immune epigenetic priming in relation to the downstream AMP-dominant skin phenotype, while also offering a psoriasis-focused, pretreatment, cell-sorted methylation resource with clearly defined healthy control and psoriasis groups. We fully acknowledge the importance of CD4+ T cells in psoriasis pathobiology; however, CD8+ T cells were prioritized here because they more directly matched the cytotoxic/tissue residency-centered systems hypothesis of the present study [35,36,37].

A dataset-wise summary of preprocessing, statistical analysis, and functional/regulatory interpretation across the Triple-Hit framework is provided in Supplementary Table S2.

### 2.2. Gut Microbiome Shotgun Metagenomic Analysis (GSE239722)

#### 2.2.1. Metagenomic Data Processing and Functional Profiling

Shotgun metagenomic sequencing data were obtained from the publicly available dataset, GSE239722, comprising 16 samples: healthy controls (HCs, *n* = 8) and patients with untreated psoriasis (PsO-UT, *n* = 8). The original study generated paired-end shotgun metagenomic reads using the Illumina NovaSeq 6000 platform. In the present analysis, we used the author-provided processed functional abundance profiles. Briefly, as reported by the data generators, reads were assembled into contigs using MEGAHIT, genes were predicted with MetaGeneMark, and a non-redundant gene catalog was constructed using MMseqs2 (95% sequence identity, 90% overlap) [38,39,40]. Functional annotation was performed against the KEGG database (E-value < 1 × 10^−5^), and gene-level abundances were aggregated to KEGG Pathway Level 3 and normalized to relative abundance [41].

#### 2.2.2. Statistical Analysis of Functional Remodeling

To characterize the gut functional landscape in psoriasis, we compared the relative abundance of metabolic pathways between groups using the Wilcoxon rank-sum test (Mann–Whitney U test), a non-parametric method suitable for the sample size (*n* = 8 per group). *p*-values were adjusted for multiple comparison using the Benjamini–Hochberg false discovery rate (FDR) procedure. We specifically interrogated pathways linked to the “Gut–Skin Axis,” focusing on Short-Chain Fatty Acid (SCFA) metabolism (e.g., Butanoate metabolism, Propanoate metabolism) and Lipid degradation pathways.

#### 2.2.3. Lipid Degradation Functional Score

To quantify the aggregate reduction in the gut’s lipid catabolic potential—the proposed “Trigger” in our Triple-Hit model—we developed a composite “Lipid Degradation Functional Score.” This score integrates three representative lipid catabolic pathways: *Fatty acid degradation (ko00071)*, *Glycerolipid metabolism (ko00561)*, and *Glycerophospholipid metabolism (ko00564)*. To ensure that pathways with varying baseline abundances contributed equally to the composite signature, we applied z-score standardization. The score for each sample j is defined as the sum of the z-scores of the constituent pathways:

$$Score_{j}\;=\;\sum_{i\;\in \;\mathrm{LipidSet}}\;Z_{ij}\;=\;\sum_{i\;\in \;\mathrm{LipidSet}}\;\frac{X_{ij}\;-\;\mu_{i}}{\sigma_{i}}$$

where $X_{ij}$ is the relative abundance of pathway i in sample j, and $\mu_{i}$ and $\sigma_{i}$ are the mean and standard deviation of pathway i across the entire cohort. Group differences in this composite score were evaluated using the Wilcoxon rank-sum test. The detailed definition of this composite score is provided in Appendix A.

### 2.3. Systemic Immune Cell DNA Methylation Analysis (GSE200376 and GSE184500)

#### 2.3.1. Shared Preprocessing and Quality Control

Publicly available genome-wide DNA methylation profiles of peripheral blood mononuclear cells (PBMCs; GSE200376) and purified CD8+ T cells (GSE184500) were generated using the Illumina Infinium MethylationEPIC BeadChip (850K). Raw intensity data (IDAT) were processed in R (version 4.5.1) using the minfi package (version 1.56) [42]. For both datasets, we applied Noob (normal-exponential out-of-band) normalization for background correction and dye-bias adjustment [43].

For downstream analysis, β values were used for biological visualization and effect-size estimation (Δβ), whereas M-values were used for statistical testing. Quality control included exclusion of probes with a detection *p*-value > 0.01 in any sample, as well as removal of probes located on sex chromosomes and probes overlapping known SNPs. Additional probe filtering steps, including exclusion of cross-reactive probes where applicable, were implemented to minimize technical bias.

#### 2.3.2. PBMC Methylome Analysis (GSE200376)

To characterize systemic immune-state–linked epigenetic remodeling in psoriasis at the aggregate circulating immune cell level, we analyzed PBMC methylation profiles from GSE200376. Because the aim of this analysis was to capture the composite systemic immune state rather than methylation changes conditional on estimated cell-type proportions, we did not regress out immune cell composition. This approach allowed the PBMC methylome signature to retain disease-associated shifts in circulating immune populations together with locus-level methylation remodeling.

Differentially methylated regions (DMRs) were identified using DMRcate with Gaussian kernel smoothing (bandwidth lambda = 1000 bp, scaling factor C = 2) [44]. Significant DMRs were defined using an FDR < 0.05 threshold. In parallel, differentially methylated positions (DMPs) were assessed at the single-CpG level using limma on M-values [45]. To reduce potential confounding by demographic factors, Sex and Age were included as covariates in the linear model design matrix (~Group + Sex + Age).

#### 2.3.3. Purified CD8+ T-Cell Methylome Analysis (GSE184500)

To examine cell-type-specific epigenetic priming in psoriasis, we analyzed methylation profiles of purified circulating CD8+ T cells from GSE184500. Because this dataset comprised a sorted immune cell population, the analysis was focused on region-based methylation remodeling within a defined cytotoxic T-cell compartment rather than on aggregate immune composition effects.

DMRs were identified using DMRcate (version 3.6.0) with the same Gaussian smoothing parameters (lambda = 1000 bp, C = 2) [44]. Significance was evaluated using the FDR of the Stouffer-transformed *p*-values (HMFDR). For exploratory pathway-level interpretation and figure-level presentation, we used a relaxed discovery threshold of FDR < 0.10 together with a mean methylation difference |Δβ| ≥ 0.02. For representative target-locus visualization, each DMR was summarized by the region-median β value across CpGs in the identified region.

#### 2.3.4. Functional Enrichment with Promoter Bias Control

To interpret the biological relevance of DMR-associated genes in both methylome datasets, we performed over-representation analysis (ORA) while accounting for the array’s strong promoter coverage bias. In both analyses, we used a custom background universe restricted to promoter-associated genes (TSS1500, TSS200, 5′UTR, and 1st Exon) rather than the whole genome.

For the PBMC dataset, ORA was used to interpret the broad DMR landscape in relation to systemic immune-state remodeling. For the purified CD8+ T-cell dataset, ORA was performed using the clusterProfiler package (version 4.18.4) [46], and DMR-associated genes were mapped to GO Biological Process, KEGG, and Reactome pathway collections. This promoter-aware strategy was used to support conservative interpretation of pathway enrichment and to reduce bias attributable to array design rather than biology.

### 2.4. Skin miRNA Expression Analysis (GSE220586)

#### 2.4.1. Data Preprocessing and Quality Control

Normalized miRNA expression profiles were obtained from GSE220586 (GPL17107; miRCURY LNA microRNA Array, 7th Gen, Exiqon/QIAGEN, Vedbaek, Denmark). To ensure robust downstream analysis, we applied stringent quality control criteria using a custom R pipeline. Features with non-finite values were converted to NA, and miRNAs were retained only if valid measurements were present in at least 6 of 8 samples (75% detection rate). Remaining missing values were imputed using row-wise median imputation to prevent artificial variance inflation. Low-variance features were removed prior to Principal Component Analysis (PCA) to ensure numerical stability.

#### 2.4.2. Differential miRNA Expression Analysis

Differential expression between psoriatic lesional skin (PsO-L) and healthy controls (HC) was assessed using the limma package (version 3.66.0). A no-intercept design matrix was constructed, and contrasts were defined as (PsO-L–HC). We applied empirical Bayes moderation (eBayes) and adjusted *p*-values using the Benjamini–Hochberg (BH) procedure. Significant differentially expressed miRNAs (DEMs) were defined based on an adjusted *p*-value (FDR) < 0.05. However, to ensure that the downstream regulatory bridge focused only on biologically potent drivers, we utilized a subset of these DEMs satisfying an additional effect size threshold of $\left|{log}_{2}\;fold-change\right|\;>\;1.0$ for visualization and network construction.

#### 2.4.3. Multi-Tiered miRNA Target Prediction and Evidence Scoring

To construct a high-confidence regulatory network relevant to lipid and AMP pathology, we implemented a multi-tiered evidence scoring system integrating predictions from multiMiR (querying TargetScan, DIANA, miRTarBase, and TarBase) and a local implementation of miRDB v6.0. Identified miRNA–target interactions were categorized into evidence tiers [47,48,49,50,51,52]:

• Level 2 (High Confidence): Interactions supported by experimentally validated evidence (miRTarBase and/or TarBase), with or without additional in silico support.
• Level 1 (Moderate Confidence): Non-validated interactions supported by at least two independent prediction resources, including multiMiR-based resources and/or miRDB.
• Level 0 (Low Confidence): Interactions supported by only a single prediction resource.

Only interactions classified as Level 1 or higher were retained for the regulatory bridge analysis, whereas single-resource predictions (Level 0) were excluded.

#### 2.4.4. Directionality-Constrained “Triple-Hit” Bridge Construction

We constructed a miRNA-mRNA regulatory bridge by integrating the high-confidence miRNA targets with the matched transcriptomic dataset (GSE186063, processed as described in Section 2.5).

1. **ID Mapping**: Ensembl IDs from the mRNA dataset were mapped to HGNC symbols using org.Hs.eg.db.
2. **Directionality Constraint**: We retained only pairs adhering to canonical repression logic: Upregulated miRNA (PsO-L) targeting downregulated mRNA, and downregulated miRNA (PsO-L) targeting upregulated mRNA.
3. **Pathological Module Filtering**: The constrained pairs were mapped to predefined “Triple-Hit” effector modules: AMP Core (Literature/DEG-driven), Barrier-Lipid Core and Keratinocyte Differentiation. This filtering strategy prioritized mechanistically interpretable links over global correlation, specifically highlighting the epigenetic control of lipid metabolism and antimicrobial defense.

### 2.5. Skin Transcriptome Analysis (GSE186063)

#### 2.5.1. Dataset and Preprocessing

Bulk RNA-sequencing data of human skin were obtained from the publicly available dataset GSE186063. Transcriptomic profiles of lesional psoriasis skin (PsO-L) were compared with those of healthy-appearing skin from patients with ankylosing spondylitis (AS-HC). Expression data were processed and analyzed using R (v4.5.1). For downstream analyses, gene-level expression values were normalized using the trimmed mean of M-values (TMM) method and transformed to log2 counts-per-million (logCPM) [53]. Sample annotations were used to define experimental groups, and only samples corresponding to AS-HC and PsO-L were retained for analyses. Gene identifiers were harmonized to Ensembl gene IDs, and gene symbols were mapped using standard human gene annotation resources from Bioconductor to enable downstream pathway, transcription factor, and cell-type analyses [54].

In this dataset, the comparator samples corresponded to healthy-appearing skin from patients with ankylosing spondylitis (AS-HC) rather than bona fide healthy control skin. Accordingly, the GSE186063 transcriptome analysis was interpreted as a within-dataset comparison between PsO-L and the available AS-HC baseline, and the robustness of the major skin-level findings was further examined in an external RNA-seq cohort containing bona fide healthy controls (GSE121212; Supplementary Figure S6).

#### 2.5.2. Differential Expression Analysis and Visualization

Differential expression analysis between PsO-L and AS-HC was performed using linear modeling implemented in the limma package (version 3.66.0). Empirical Bayes moderation was applied to stabilize variance estimates across genes. Differentially expressed genes (DEGs) were identified based on log2 fold-change and moderated statistics, with *p* values adjusted for multiple testing using the Benjamini–Hochberg false discovery rate (FDR) procedure. DEG results were visualized using volcano plots (log2 fold-change versus −log10 adjusted *p* value), and expression patterns of selected DEGs were displayed as heatmaps, with expression values standardized by row-wise z-score for visualization [45,55].

#### 2.5.3. Hallmark Gene Set Enrichment Analysis and Axis-Level Aggregation

To characterize pathway-level transcriptional remodeling in psoriatic lesions, Gene Set Enrichment Analysis (GSEA) was performed using a pre-ranked approach. Genes were ranked according to log2 fold-change values derived from the limma analysis, preserving both effect size and directionality [56]. Enrichment analysis was conducted against the MSigDB Hallmark gene set collection. Enrichment results were summarized using normalized enrichment scores (NES), and statistical significance was evaluated using the FDR-adjusted *p* value (p.adjust, hereafter referred to as padj) [57,58].

For higher-level interpretation, Hallmark gene sets were further grouped into predefined biological axes—lipid metabolism, insulin/metabolic signaling, and inflammation—based on functional annotation rules (see Appendix B for the detailed Hallmark gene set aggregation logic). For each axis, NES values were summarized at the axis level, and axis-level significance was assessed by the median padj of the constituent Hallmark gene sets.

#### 2.5.4. Transcription Factor Activity Inference

Upstream regulatory activity was inferred by estimating transcription factor (TF) activities from gene expression data using the decoupleR framework in conjunction with curated human regulons from the DoRothEA database [59]. Regulons with high to intermediate confidence levels were used. TF activity scores were computed using a weighted mean method with permutation-based estimation. Differential TF activity between PsO-L and AS-HC was assessed using linear modeling, and results were summarized with effect sizes and FDR-adjusted *p* values for visualization [60].

#### 2.5.5. Immune and Stromal Cell Score Estimation (MCP-Counter)

To estimate the relative enrichment of immune and stromal cell populations within the skin transcriptome, cell-type scores were computed using MCP-counter based on gene expression values mapped to human gene symbols. MCP-counter scores were calculated for each sample and summarized by cell type [61]. Statistical differences in cell-type scores between PsO-L and AS-HC were assessed using the Wilcoxon rank-sum test for each cell type, with *p* values adjusted for multiple testing using the Benjamini–Hochberg FDR method. Cell-type enrichment patterns were visualized using group comparison boxplots.

### 2.6. Statistical Analysis and Visualization

All statistical analyses were conducted in R [62]. Unless otherwise stated, statistical tests were two-sided, and *p* values were adjusted for multiple comparisons using the Benjamini–Hochberg procedure. Data visualization was performed using ggplot2 and ComplexHeatmap, with consistent formatting, font sizes, and a fixed group order applied across all figures [63,64]. 

Generative AI tools were used only for debugging, refactoring, and optimization of custom R scripts for multi-omics data processing and visualization, and for English-language review and editing of the manuscript. They were not used to generate data, perform statistical analyses, interpret biological results, or formulate scientific conclusions.

## 3. Results

### 3.1. Gut Microbial Functional Remodeling in Psoriasis Indicates Reduced Lipid Catabolic Potential and Heterogeneous Shifts in SCFA-Related Pathways

To characterize gut microbial functional remodeling in psoriasis, we compared KEGG Pathway Level 3 relative-abundance profiles derived from shotgun metagenomic data between healthy controls (HC) and untreated psoriasis samples (PsO-UT). In this context, the inferred pathway profile reflects community-level functional potential, i.e., the relative representation of microbial pathways within the community, rather than direct measurement of metabolite concentrations.

At the compositional level, genus-level profiles showed marked inter-individual heterogeneity rather than a uniform disease-specific taxonomic pattern, with no clear evidence of a consistent loss of alpha diversity or a significant global shift in beta diversity (Supplementary Figures S1–S3).

**Figure 2:**
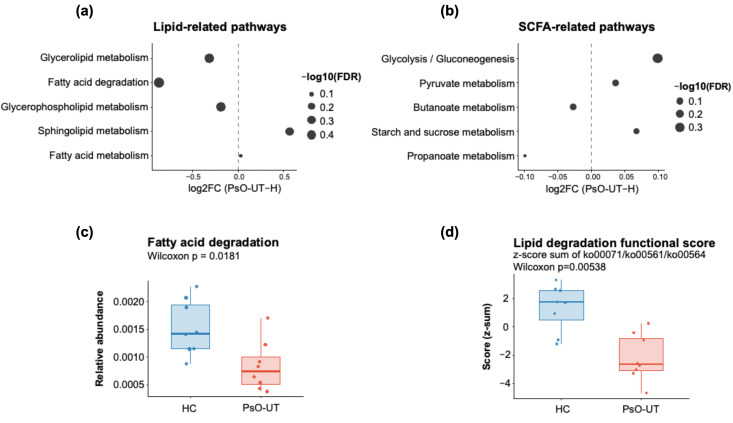
Gut microbial functional remodeling in psoriasis indicates reduced lipid catabolic potential and heterogeneous shifts in SCFA-related pathways: Shotgun metagenomic functional profiles from GSE239722 were compared between healthy controls (HC) and untreated psoriasis samples (PsO-UT) (**a**) Lipid-related pathways show an overall downward shift in PsO-UT, including reduced fatty acid degradation, whereas sphingolipid metabolism shifts upward; (**b**) SCFA-related pathways display heterogeneous directionality, with downward shifts in propanoate and butanoate metabolism but relative upward shifts in upstream carbohydrate/central carbon metabolism; (**c**) Fatty acid degradation is reduced in PsO-UT; (**d**) A composite Lipid Degradation Functional Score is also reduced in PsO-UT, supporting coordinated depletion of gut microbial lipid catabolic capacity in psoriasis.

Among lipid-associated pathways, PsO-UT showed relative downward shifts in fatty acid degradation as well as in additional lipid metabolic pathways, including glycerolipid metabolism and glycerophospholipid metabolism (Figure 2a). In contrast, sphingolipid metabolism showed an upward shift, suggesting remodeling of gut microbial lipid-related functional capacity in psoriasis, with reduced lipid catabolic potential accompanied by selective increases in specific lipid-associated processes.

SCFA-related pathways also showed a heterogeneous pattern (Figure 2b). Pathways directly linked to terminal SCFA metabolism, most notably propanoate metabolism and butanoate metabolism, showed relative downward shifts in PsO-UT. In contrast, pathways related to upstream carbohydrate utilization and central carbon metabolism, including glycolysis/gluconeogenesis, pyruvate metabolism, and starch and sucrose metabolism, showed relative upward shifts. Together, these results are consistent with a functional reconfiguration in which upstream carbohydrate/central carbon metabolism is shifted upward, whereas terminal SCFA-associated pathways show relative downward or discordant directionality in psoriasis.

To further examine representative pathway-level signals and a composite summary metric, we visualized selected readouts using box plots. Fatty acid degradation was significantly reduced in PsO-UT compared with HC (Figure 2c), consistent with lower gut microbial lipid catabolic potential in psoriasis. To summarize this pattern beyond a single pathway, we calculated a Lipid Degradation Functional Score for each sample as the sum of z-scores across three representative KEGG L3 lipid pathways (ko00071, fatty acid degradation; ko00561, glycerolipid metabolism; ko00564, glycerophospholipid metabolism). This composite score was significantly lower in PsO-UT than in HC (Figure 2d), supporting a coordinated reduction in lipid degradation-related functional capacity at the community level.

Overall, these results support a psoriasis-associated gut functional signature characterized by reduced lipid catabolic potential and heterogeneous shifts across SCFA-related pathways. Within the Triple-Hit framework, this gut layer was interpreted as a plausible upstream metabolic component linked to downstream systemic and cutaneous multi-omics features.

### 3.2. Systemic PBMC DNA Methylation Remodeling in Psoriasis (GSE200376) Highlights Widespread DMRs and Immune State-Linked Epigenetic Priming

**Figure 3:**
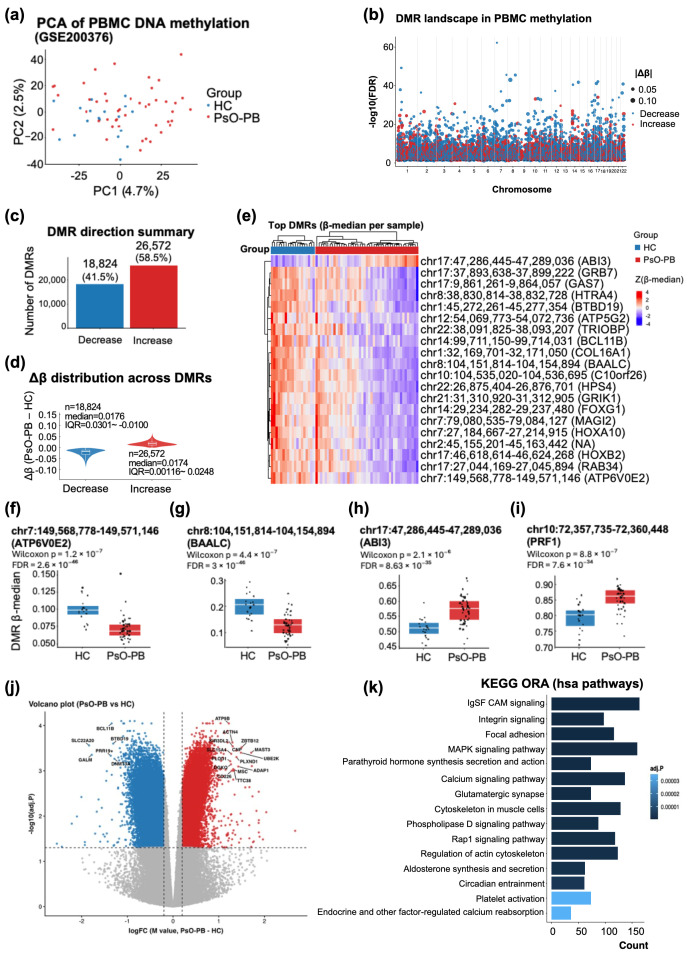
Systemic PBMC DNA methylation remodeling in psoriasis (GSE200376) reveals widespread DMRs, a reproducible top-DMR signature, and enrichment of membrane-proximal signaling programs (HC vs. PsO-PB): (**a**) samples (PsO-PB) and healthy controls (HCs), consistent with heterogeneous methylome remodeling; (**b**) Manhattan-like genome-wide DMR landscape demonstrating that significant differentially methylated regions (DMRs) are broadly distributed across essentially all chromosomes; (**c**) the summary of DMR directionality (hypermethylation vs. hypomethylation) indicating the relative burden of regions showing increased or decreased methylation in PsO-PB compared with HCs; (**d**) the distribution of regional effect sizes (Δβ) across DMRs shown separately for hypo- and hypermethylated regions, illustrating predominantly modest but consistent shifts; (**e**) a heatmap of top-ranked DMRs, where each DMR is summarized by the per-sample median β value across CpGs within the region, revealing clustering of samples by disease status; (**f**–**i**) representative DMR examples visualized as per-sample median β values for selected loci (*ATP6V0E2*, *BAALC*, *ABI3*, and *PRF1*), highlighting consistent group-level methylation shifts at individual regions; (**j**) the volcano plot of differentially methylated positions (DMPs) at the single-CpG level, highlighting CpG sites with large effect sizes and strong multiple testing-adjusted significance; (**k**) the KEGG pathway over-representation analysis (ORA) of DMR-annotated genes (hsa pathways), identifying enrichment of adhesion/cytoskeletal remodeling and membrane-associated signaling pathways consistent with systemic immune state-linked epigenetic priming.

We next profiled systemic epigenetic alterations in peripheral blood mononuclear cells (PBMCs) using EPIC 850K DNA methylation data (GSE200376; HC, *n* = 19; PsO-PB, *n* = 20). Unsupervised PCA based on highly variable CpG sites revealed a modest but discernible separation between PsO-PB and healthy controls, consistent with broad yet heterogeneous methylome remodeling rather than a single dominant axis of variation (PC1 = 4.7% and PC2 = 2.5% explained variance; Figure 3a).

Region-based analysis identified a large burden of differentially methylated regions (DMRs) distributed across the genome. The Manhattan-like landscape showed that significant DMRs were detected across essentially all chromosomes, with numerous regions exceeding the predefined false discovery rate threshold (FDR < 0.05), indicating system-wide rather than locus-restricted methylation perturbations (Figure 3b). Notably, DMR directionality was skewed toward increased methylation in psoriasis PBMCs: 26,572 DMRs (58.5%) showed increased methylation, whereas 18,824 (41.5%) showed decreased methylation (total 45,396 DMRs; Figure 3c). Although the directionality differed, the effect sizes were comparably modest in magnitude, with median Δβ of −0.0172 (IQR −0.0301 to −0.0100) for decreased-methylation DMRs and +0.0174 (IQR +0.0116 to +0.0246) for increased-methylation DMRs (Figure 3d). This pattern suggests a coordinated accumulation of small but consistent regional shifts affecting a large fraction of the methylome.

To determine whether top DMRs capture a reproducible group-level signature, we summarized each DMR by the per-sample median β value across CpGs in the region and visualized the top-ranked DMR set. The heatmap of top DMRs revealed clear clustering of samples by disease status (Figure 3e), supporting the presence of a robust systemic epigenetic signature in psoriasis PBMCs despite the modest overall separation observed in PCA. Several top DMRs were annotated to immune-state and signaling-related genes, including *BCL11B* and multiple loci linked to cellular signaling/structure (e.g., *TRIOBP* and *GRB7*), as well as developmental regulators (*HOXA10*, *HOXB2*), collectively consistent with an altered immune cell state and/or PBMC composition shift in psoriasis.

Representative DMRs further showed consistent β-median shifts between groups at the individual-region level (Figure 3f–i), recapitulating the bidirectional nature of methylation remodeling observed in the global summaries. Specifically, DMRs annotated to *ATP6V0E2* (chr7: 149,568,778–149,571,146; Wilcoxon *p* = 1.2 × 10^−7^) and *BAALC* (chr8: 104,151,814–104,154,894; Wilcoxon *p* = 4.4 × 10^−7^) exhibited lower region-level median β values in PsO-PB relative to HC, consistent with regional hypomethylation in psoriasis PBMCs. In contrast, DMRs mapped to *ABI3* (chr17: 47,286,445–47,289,036; Wilcoxon *p* = 2.1 × 10^−6^) and *PRF1* (chr10: 72,357,735–72,360,448; Wilcoxon *p* = 8.8 × 10^−7^) showed higher median β values in PsO-PB, exemplifying region-level hypermethylation at immune-relevant loci. Together, these locus-level examples illustrate that psoriasis PBMC remodeling is not confined to a single direction of change, but instead comprises coordinated, regionally structured shifts spanning both hypo- and hypermethylated programs.

At the single-CpG level (DMP analysis), the volcano plot highlighted CpG sites jointly exhibiting large effect sizes and strong multiple-testing–adjusted significance (Figure 3j). Labeled hits included CpGs annotated to immune lineage/effector modules (*BCL11B*, *KIR3DL2*, and *CD226*), an epigenetic regulator (*DNMT3A*), and metabolic or membrane-associated signaling genes (*DGKQ*, *GALM*, *SLC15A4*, *PLOD1*, and *PLXND1*), suggesting that systemic psoriasis may involve coordinated perturbations spanning immune-state regulation, epigenetic machinery, and membrane-proximal signaling axes.

To contextualize the DMR signal at the pathway level, we performed KEGG pathway over-representation analysis (ORA; hsa pathways) on DMR-annotated genes. Enriched terms prominently included adhesion and cytoskeletal remodeling programs (e.g., IgSF CAM signaling, integrin signaling, focal adhesion) together with membrane-associated signal transduction pathways (e.g., MAPK signaling, calcium signaling, Rap1 signaling, phospholipase D signaling) (Figure 3k). Collectively, these results support a model in which psoriasis is associated with widespread PBMC epigenome remodeling characterized by a predominance of hypermethylated regions, accompanied by a reproducible top-DMR signature and prominent CpG-level alterations mapping to immune identity and membrane-proximal signaling programs consistent with systemic immune-state–linked epigenetic priming.

### 3.3. Directional DNA Methylation Remodeling of Circulating CD8^+^ T Cells in Psoriasis Reveals a Hypomethylation-Biased Regional Profile Enriched for Lipid- and Membrane-Associated Pathways (GSE184500)

**Figure 4:**
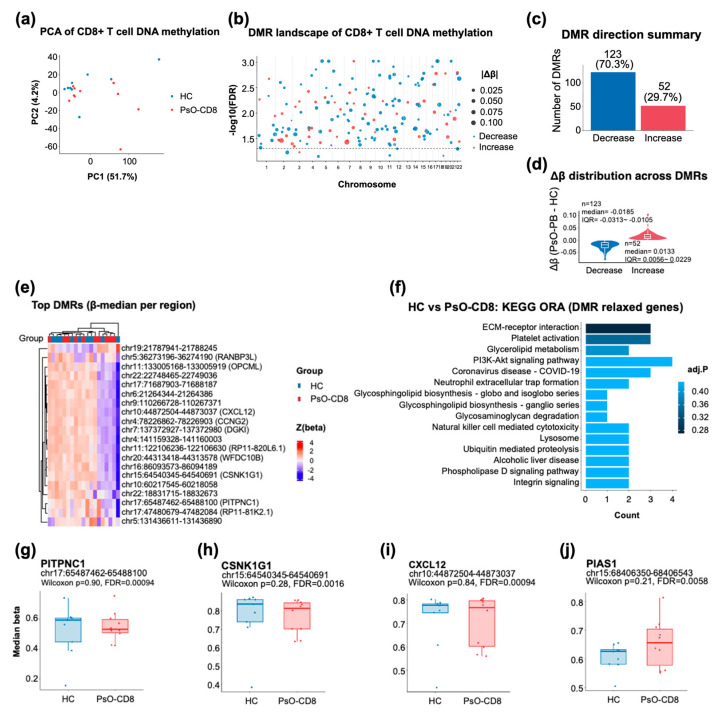
Directional DNA methylation remodeling of circulating CD8^+^ T cells in psoriasis (GSE184500): (**a**) principal component analysis (PCA) of genome-wide DNA methylation profiles (Illumina MethylationEPIC) from purified circulating CD8^+^ T cells comparing healthy controls (HCs, *n* = 9) and psoriasis before treatment (PsO-CD8, *n* = 10). The PCA was computed using highly variable CpG sites (β values after preprocessing); the points are colored by group (HC, blue; PsO-CD8, red) and show a modest group-level separation; (**b**) a genome-wide DMR landscape identified through region-based analysis (DMRcate). Each point represents a DMR plotted across chromosomes; the *y*-axis indicates −log10(FDR) for the DMR, the point size reflects |Δβ| (PsO-CD8 − HC; region-median β), and color denotes direction (decrease vs. increase in PsO-CD8 relative to HC); (**c**) a directionality summary of detected DMRs, showing that most regions exhibit decreased methylation in PsO-CD8 relative to HC (123/175, 70.3%), compared with increased methylation (52/175, 29.7%); (**d**) the distribution of Δβ across DMRs stratified by direction. Violin/box plots summarize region-median methylation differences (Δβ), with median (IQR) of −0.0185 (−0.0313 to −0.0105) for decreased DMRs (*n* = 123) and 0.0133 (0.0056 to 0.0229) for increased DMRs (*n* = 52); (**e**) a heatmap of top-ranked DMRs showing region-median β values (row z-score) with samples ordered HC → PsO-CD8; the rows are annotated by genomic coordinates and representative overlapping gene symbols where available; (**f**) the KEGG pathway over-representation analysis (ORA) for genes linked to the relaxed DMR set used in this figure. Top-ranked pathways include membrane/ECM and signaling-associated terms (e.g., ECM–receptor interaction, PI3K–Akt signaling), along with lipid- and glyco-conjugate–related pathways (e.g., glycerolipid metabolism; glycosphingolipid biosynthesis); (**g**–**j**) representative DMR loci visualized as boxplots of region-median β values with individual samples overlaid: PITPNC1 (**g**), CSNK1G1 (**h**), CXCL12 (**i**), and PIAS1 (**j**). Each panel reports the Wilcoxon rank-sum *p* value for the group comparison and the corresponding DMR-level FDR from the region-based analysis (as labeled in the plots); a positive Δβ indicates higher methylation in PsO-CD8 relative to HC.

To assess whether circulating effector T cells exhibit disease-associated epigenetic remodeling in psoriasis, we analyzed genome-wide DNA methylation profiles of purified CD8^+^ T cells generated using the Illumina MethylationEPIC array (GSE184500), comparing healthy controls (HC, *n* = 9) and psoriasis patients before treatment (PsO-CD8, *n* = 10). Unsupervised principal component analysis (PCA) based on highly variable CpG sites showed a modest but discernible separation between PsO-CD8 and HC, indicating a global shift in the CD8^+^ T-cell methylation landscape associated with psoriasis (Figure 4a).

We next performed region-based analysis using DMRcate to identify coordinated methylation changes across adjacent CpG sites. Differentially methylated regions (DMRs) were distributed across multiple chromosomes rather than confined to a limited number of loci, consistent with widespread but regionally structured epigenetic remodeling (Figure 4b). In total, 175 DMRs were detected using the criteria applied in this figure. Notably, these regions exhibited a pronounced directional bias: 123 DMRs (70.3%) showed decreased methylation in PsO-CD8 relative to HC, whereas 52 DMRs (29.7%) showed increased methylation (Figure 4c). Examination of effect-size distributions further demonstrated that hypomethylated and hypermethylated DMRs occupied distinct Δβ ranges, with region-median Δβ values centered at −0.0185 (IQR −0.0313 to −0.0105) for decreased DMRs and 0.0133 (IQR 0.0056 to 0.0229) for increased DMRs (Figure 4d). Together, these results indicate a hypomethylation-biased pattern within the set of regions undergoing differential methylation in circulating CD8^+^ T cells in psoriasis.

A heatmap of top-ranked DMRs based on region-median β values further illustrated coherent group-level methylation patterns when samples were ordered HC → PsO-CD8 (Figure 4e). Although effect sizes at individual regions were modest, the consistency of directional changes across multiple loci supports the presence of structured epigenetic remodeling rather than stochastic variation.

To explore the functional themes represented by DMR-associated genes, we conducted KEGG pathway over-representation analysis using a relaxed DMR discovery set, as specified for this figure. The top-ranked pathways included modules related to membrane organization and signaling, such as ECM–receptor interaction and PI3K–Akt signaling, alongside lipid- and glyco-conjugate–related processes including glycerolipid metabolism and glycosphingolipid biosynthesis (Figure 4f). While these enrichments did not uniformly reach stringent significance thresholds, their convergence on lipid-associated and membrane-proximal pathways suggests that regional DNA methylation changes in circulating CD8^+^ T cells preferentially affect programs relevant to cellular structure, signaling, and metabolic adaptation.

To provide locus-level context, we visualized region-median methylation patterns for four representative DMRs selected for biological interpretability: *PITPNC1*, *CSNK1G1*, *CXCL12*, and *PIAS1* (Figure 4g–j). These loci span functions related to lipid-associated signaling and trafficking (*PITPNC1*), kinase-mediated regulatory pathways (*CSNK1G1*), immune cell migration (*CXCL12*), and SUMOylation-linked control of inflammatory signaling (*PIAS1*). Boxplots illustrate the distribution of region-median β values across groups, with individual sample values overlaid. While group-wise differences at single loci were variable—consistent with modest effect sizes and limited sample numbers—each region was identified as a DMR by region-based analysis, supporting their inclusion as illustrative examples of the broader epigenetic remodeling observed.

Collectively, these results demonstrate that circulating CD8^+^ T cells in psoriasis exhibit a directionally biased pattern of regional DNA methylation changes, characterized by a predominance of hypomethylated DMRs and lipid- and membrane-associated functional themes. Rather than indicating direct transcriptional activation at specific loci, this pattern is consistent with an epigenetically primed cellular state linked to altered immune cell regulatory potential.

### 3.4. Lesional miRNA Remodeling in Psoriasis and Directionality-Constrained miRNA–mRNA Bridging Aligns with AMP Activation and Barrier–Lipid/Keratinocyte Differentiation Modules (GSE220586)

**Figure 5:**
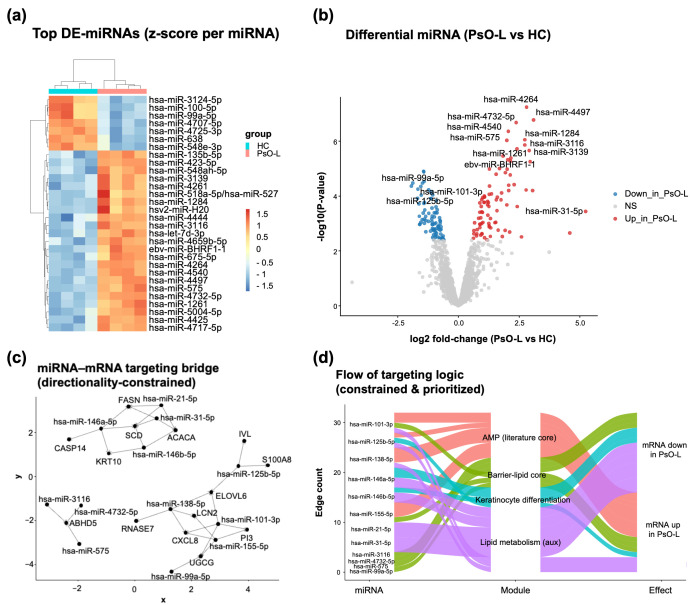
Lesional miRNA remodeling and directionality-constrained miRNA–mRNA bridging highlight links to AMP programs and barrier–lipid/keratinocyte differentiation modules in psoriasis: (**a**) a heatmap of the top differentially expressed miRNAs between healthy controls (HCs, *n* = 4) and psoriatic lesional samples (PsO-L, *n* = 4). The values are shown as a row-scaled expression (z-score) to emphasize relative up/down patterns across samples; hierarchical clustering separates HCs and PsO-L; (**b**) volcano plot summarizing differential miRNA expression in PsO-L versus HC. The *x*-axis indicates a log2 fold change (PsO-L vs. HC) and the *y*-axis indicates −log10(*p* value). miRNAs meeting the significance threshold (FDR < 0.05) are considered differentially expressed, and selected miRNAs with large effect sizes and strong statistical support are labeled; (**c**) directionality-constrained miRNA–mRNA targeting bridge network. Candidate miRNA–target pairs were compiled from miRNA target evidence resources and prioritized using the evidence-filtering strategy described in Methods, and only pairs showing inverse miRNA/mRNA directionality (miRNA ↑/mRNA ↓ or miRNA ↓/mRNA ↑) were retained, consistent with canonical miRNA-mediated repression. Nodes represent miRNAs and their putative target genes, and edges indicate retained miRNA-to-target links; (**d**) Alluvial (Sankey) summary of the constrained bridge, showing how prioritized miRNAs connect to functionally curated target modules (AMP literature core, barrier–lipid core, keratinocyte differentiation, and auxiliary lipid metabolism) and to the observed direction of the target mRNA change in PsO-L. Flow width represents the number of retained miRNA–mRNA edges (edge count) after directionality filtering and prioritization.

To characterize post-transcriptional regulatory remodeling in psoriatic lesions, we analyzed lesional miRNA expression profiles from GSE220586 (PsO-L, *n* = 4; HC, *n* = 4). Unsupervised clustering of the top differentially expressed miRNAs revealed clear separation between PsO-L and HC, indicating that a compact miRNA signature captures a coherent lesional regulatory state (Figure 5a). Differential expression analysis identified 168 significant miRNAs (FDR < 0.05; 86 upregulated and 82 downregulated in PsO-L vs. HC), and the volcano summary highlighted multiple high-effect signals across both directions (Figure 5b).

To translate miRNA changes into mechanistically interpretable downstream consequences, we integrated lesional miRNA signals with lesional skin mRNA changes using a directionality-constrained miRNA–mRNA bridging strategy, retaining only pairs showing inverse directionality (miRNA ↑/mRNA ↓ or miRNA ↓/mRNA ↑), consistent with canonical miRNA-mediated repression (Figure 5c). This constraint reduces ambiguity inherent to target prediction by prioritizing interactions that are internally consistent within the paired miRNA and mRNA datasets.

The resulting constrained bridge network connected prioritized miRNAs to targets spanning biologically coherent modules that represent hallmark features of psoriatic lesions (Figure 5c). In particular, retained targets mapped to (i) an AMP literature core, (ii) a barrier–lipid core, and (iii) a keratinocyte differentiation module, with additional links to auxiliary lipid metabolism (Figure 5d). The alluvial summary further visualized how the retained miRNA–mRNA edges converge on these modules and align with the observed direction of target mRNA dysregulation in PsO-L (Figure 5d). Collectively, these analyses support that lesional miRNA reprogramming forms a directionally coherent regulatory layer that is closely aligned with cutaneous AMP activation and barrier–lipid/keratinocyte differentiation remodeling in psoriasis.

### 3.5. Lesional Skin Transcriptomics in Psoriasis Reveals an Inflammatory–Proliferative State with Coordinated TF Activity Shifts and Immune Cell Signature Remodeling

**Figure 6:**
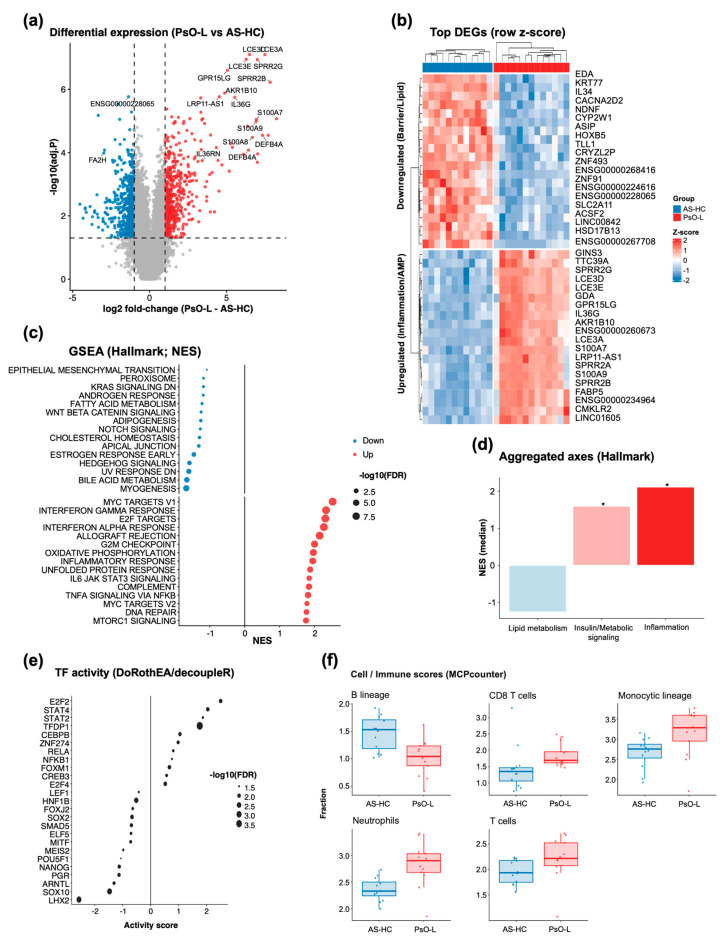
Transcriptomic remodeling in psoriatic lesional skin highlights an inflammatory–proliferative program with altered metabolic axes, coordinated TF activity shifts and immune cell signature differences; (**a**) differential expression volcano plot comparing PsO-L and AS-HC. The *x*-axis indicates log2 fold change (PsO-L − AS-HC) and the *y*-axis indicates −log10 (adjusted *p* value). Dashed lines denote the significance and fold change thresholds used for DEG classification (|log2FC| ≥ 1 and FDR ≤ 0.05); (**b**) a heatmap of top differentially expressed genes (DEGs) across samples, visualized as row-wise z-scores. The genes are displayed as representative upregulated and downregulated sets in PsO-L relative to AS-HC; (**c**) the Hallmark gene set enrichment analysis (GSEA) performed on a ranked gene list derived from differential expression results, summarized by normalized enrichment score (NES). Dot size reflects −log10(FDR), and the direction indicates an enrichment in PsO-L (positive NES) or AS-HC (negative NES); (**d**) the aggregated axis summary of Hallmark GSEA results, in which gene sets are grouped into predefined biological axes and summarized as axis-level NES values. In panel (**d**), asterisks indicate aggregated axes in which the median FDR-adjusted *p* value (padj) of the constituent Hallmark gene sets was <0.05; (**e**) the transcription factor (TF) activity inference using DoRothEA regulons and decoupleR (weighted mean), reporting differential TF activity between PsO-L and AS-HC and visualizing top signals with effect size and FDR-based significance encoding; (**f**) the cell/immune enrichment scores estimated from bulk transcriptomes using MCP-counter and compared between AS-HC and PsO-L; the group differences were assessed using the Wilcoxon rank-sum test with Benjamini–Hochberg FDR correction.

To characterize psoriasis-associated transcriptional programs in skin, we compared bulk RNA-seq profiles of psoriatic lesional skin (PsO-L) against the available within-dataset comparator, healthy-appearing skin from patients with ankylosing spondylitis (AS-HC). Differential expression analysis revealed a strong disease signature dominated by keratinocyte activation/barrier remodeling and inflammation-related genes, with prominent upregulation of canonical psoriatic markers including *DEFB4A*, *S100A7/A8/A9*, and *IL36G* (Figure 6a). A heatmap of the top differentially expressed genes demonstrated clear separation of PsO-L from AS-HC, indicating a coherent lesion-specific expression program (Figure 6b).

At the pathway level, Hallmark gene set enrichment analysis showed robust enrichment of inflammatory and immune signaling in PsO-L, alongside proliferative cell-cycle programs (Figure 6c). Enriched signatures included high-NES pathways such as TNF/NF-κB–linked inflammation, IL6–JAK–STAT signaling, complement and Interferon-associated responses, and cell-cycle regulators (e.g., *E2F TARGETS*, *G2M CHECKPOINT*), consistent with an activated inflammatory–hyperproliferative lesional phenotype. In contrast, gene sets reflecting epithelial differentiation/homeostatic features and selected metabolic programs, such as *FATTY ACID METABOLISM*, trended toward AS-HC enrichment (negative NES). To facilitate an interpretable summary, we aggregated Hallmark enrichments into three keyword-defined conceptual axes based on keyword matching of Hallmark gene set name: Inflammation (IL-17, TNF, Interferon, NF-kB, inflamm), Lipid metabolism (lipid, fatty, sphingo, cholesterol), and Insulin/Metabolic signaling (insulin, PI3K, AKT, mTOR, Glycolysis). This axis-level aggregation indicated a relative downshift of lipid-metabolism–related Hallmark programs in PsO-L accompanied by concurrent increases in inflammation- and insulin/metabolic signaling–related programs (Figure 6d), supporting coordinated inflammatory–metabolic reprogramming within lesional skin. Single-sample Hallmark activity profiles further supported the same lesion-level directionality for representative inflammatory and metabolic pathways (Supplementary Figure S4).

To assess whether these skin-level conclusions depended on the use of AS-HC as the available comparator in GSE186063, we additionally analyzed an independent psoriasis skin RNA-seq cohort containing bona fide healthy controls (GSE121212; Supplementary Figure S6). In this external validation cohort, PsO-L again showed strong induction of canonical lesional inflammatory genes centered on IL36/S100/defensin-associated programs, together with Hallmark-level enrichment of inflammatory pathways and relative reduction in homeostatic/barrier-like programs. These findings indicate that the major inflammatory and AMP-associated transcriptomic conclusions of the GSE186063 analysis are not solely dependent on the AS-HC control definition, although the magnitude of selected metabolic/lipid aggregates may vary across cohorts and comparator contexts.

We next inferred upstream regulatory activity using DoRothEA regulons and decoupleR. TF activity analysis identified a set of transcriptional regulators with increased inferred activity in PsO-L, driven by top-ranked factors such as *E2F2*, *STAT4/2*, and *RELA* (NF-κB subunit), whereas a subset of TFs, including *LHX2* and *SOX10*, displayed decreased inferred activity (Figure 6e). Finally, immune/stromal signature deconvolution using MCP-counter revealed distinct alterations in cellular composition between PsO-L and AS-HC (Figure 6f). Correlation analysis further linked AMP gene expression to inferred immune/stromal abundance patterns, providing orthogonal support for the relationship between the AMP-dominant lesional program and the inflammatory cellular context (Supplementary Figure S5). Specifically, we observed significant enrichment of key effector cells involved in psoriasis pathogenesis, including neutrophils (FDR = 0.008), CD8 T cells (FDR = 0.015), and overall T cell populations (FDR = 0.044). The monocytic lineage (FDR = 0.029), associated with inflammatory responses, was also significantly upregulated. In contrast, B lineage scores were significantly reduced in PsO-L compared to AS-HC (FDR = 0.015). Collectively, these results position lesional psoriasis skin as an inflammatory–proliferative state with coordinated TF-level control and an accompanying shift in keyword-defined metabolic axes, providing transcriptomic support for downstream multi-omics integration.

## 4. Discussion

In this study, we applied an integrative multi-omics framework to examine coordinated molecular alterations across the gut–blood–skin axis in psoriasis, focusing on the coordinated interplay between gut microbial functional remodeling, immune cell epigenetic reprogramming, and lesional skin transcriptional activation. By analyzing independent public datasets spanning gut metagenomics, DNA methylation in circulating and cell-sorted immune populations, and skin transcriptomics, we provide convergent evidence that psoriasis is characterized not by isolated molecular perturbations, but by a convergent remodeling axis that links microbial metabolic capacity, immune cell epigenetic priming, and an AMP-dominant cutaneous inflammatory program.

A central finding of this work is the observation that psoriasis-associated gut dysbiosis is accompanied by a selective reduction in the relative abundance of microbial lipid catabolic and SCFA-associated metabolic pathways, rather than a uniform loss of metabolic capacity. Functional profiling of shotgun metagenomic data revealed consistent downward shifts in pathways related to fatty acid degradation and short-chain fatty acid metabolism—including butanoate and propanoate metabolism—alongside compensatory enrichment of alternative lipid-associated processes such as sphingolipid metabolism. These results are consistent with previous reports of reduced fecal SCFA levels in psoriasis [8,65] but specifically highlight that the disturbance is imprinted at the level of microbial functional potential, suggesting an ecological shift in metabolic capacity rather than simply reflecting transient metabolite depletion. Importantly, this functional imbalance is compatible with an upstream context that could plausibly contribute to host immune regulation through well-established SCFA-mediated epigenetic and immunomodulatory mechanisms [66,67,68].

At the systemic immune level, our methylome analyses of PBMCs and purified CD8+ T cells revealed widespread DNA methylation remodeling in psoriasis, characterized by distributed differentially methylated regions (DMRs) across the genome. The identification of coherent DMR landscapes, rather than isolated differentially methylated positions, underscores that psoriasis is associated with coordinated epigenetic reprogramming of immune cells [14,15,16,69]. Functional enrichment of DMR-associated genes highlighted pathways involved in immune activation, signal transduction, and membrane-proximal regulatory processes. Notably, the epigenetic remodeling of lipid-associated signaling loci in CD8+ T cells (e.g., PITPNC1) conceptually resonates with the observed loss of gut microbial lipid catabolic capacity, supporting the plausibility of a systemic metabolic–epigenetic axis in which pathway-level themes co-occur across compartments.

The CD8+ T cell–specific methylation analysis further strengthens this interpretation. Despite the limited sample size inherent to cell-sorted methylome datasets, the detection of structured DMR landscapes and functionally coherent pathway enrichment indicates that cytotoxic T cells in psoriasis undergo targeted epigenetic reprogramming. These changes may contribute to altered effector differentiation, cytotoxic potential, and tissue residency, all of which are critical features of psoriatic inflammation [70,71]. The use of a relaxed DMR discovery threshold for pathway-level over-representation analysis allowed recovery of biologically meaningful signals while maintaining transparency regarding statistical stringency, a balance that is increasingly recognized as necessary for functional interpretation of epigenomic data in modestly sized cohorts.

At the tissue level, lesional skin transcriptome analysis revealed a pronounced AMP-dominant inflammatory program, accompanied by activation of canonical immune and stress-response pathways [72,73]. Crucially, our integration of lesional miRNA profiles (GSE220586) provides a mechanistically interpretable layer to this transcriptional dysregulation. The identified directionality-constrained miRNA–mRNA bridge suggests that the downregulation of key regulatory miRNAs is consistent with reduced post-transcriptional restraint on inflammatory targets, effectively reinforcing the AMP-dominant state [26,74]. By integrating these post-transcriptional findings with upstream immune methylation and gut functional alterations, our data suggest that the lesional transcriptome reflects not only local cytokine-driven inflammation but also the downstream manifestation of systemic immune priming. In this context, the skin emerges as an effector organ responding to a “primed” immune landscape rather than the sole origin of disease activity [1,2].

Importantly, immune cell infiltration analysis using MCP-counter provided orthogonal support for this model. The increased abundance scores of cytotoxic lymphocytes, myeloid lineages, and other immune populations in psoriatic lesions align with the observed transcriptomic activation and reinforce the biological relevance of the inferred immune signatures [20]. While MCP-counter does not quantify absolute cell numbers, its marker-based abundance estimates offer a robust means of contextualizing bulk RNA-seq data and linking transcriptional patterns to changes in cellular composition.

Taken together, these findings support a model in which psoriasis arises from the convergence of three interrelated hits: (i) Putative Trigger: Gut microbial functional remodeling characterized by reduced genomic potential for lipid catabolism and SCFA metabolism, (ii) Putative Mediator: Systemic immune cell epigenetic priming involving coordinated DNA methylation changes linked to lipid/membrane signaling, and (iii) Downstream Effector: Amplification of an AMP-driven inflammatory program in the skin, stabilized by disrupted miRNA-mediated post-transcriptional control. This “Triple-Hit” framework provides a conceptual scaffold and mechanistic hypothesis bridging microbial ecology, immune regulation, and tissue pathology, offering a systems-level perspective that complements and extends existing cytokine-centric models of psoriasis [21,75].

For example, reduced gut microbial lipid catabolic and SCFA-associated functional potential (GSE239722) was interpreted together with lipid/glycosphingolipid-associated methylation changes in circulating immune cells (GSE184500) and an AMP-dominant lesional effector program in skin (GSE186063/GSE220586), thereby linking upstream metabolic remodeling, systemic immune priming, and downstream cutaneous inflammatory amplification at the level of biological theme alignment rather than direct sample matching.

Several limitations should be acknowledged. All analyses were conducted using independent publicly available datasets, precluding direct experimental validation or longitudinal assessment within the same individuals. In addition, inferred microbial functions and immune cell abundance scores represent indirect estimates rather than direct measurements.

An additional limitation of this study is incomplete age metadata across the public cohorts. Age information was unavailable for GSE239722, GSE220586, and GSE121212, precluding the implementation of a uniform age-adjusted model across the full multi-omics framework. Accordingly, residual age-related confounding cannot be excluded, particularly in cross-cohort interpretation. Although within-dataset contrasts were used throughout and covariate-aware modeling was applied where metadata availability permitted, these strategies reduce but do not eliminate potential age effects.

A further limitation concerns the transcriptomic comparator used in GSE186063. In that cohort, the available comparator consisted of healthy-appearing skin from patients with ankylosing spondylitis (AS-HC), which cannot be considered equivalent to bona fide disease-free skin because psoriasis is a recognized extra-musculoskeletal manifestation within the spondyloarthritis spectrum. At the same time, because the comparator in GSE186063 may carry a low-level inflammatory background related to the spondyloarthritis spectrum, the lesional-versus-comparator contrast may have been conservative rather than artificially inflated; thus, inflammatory and AMP-dominant signals that remained prominent against this background are less likely to be explained solely by trivial comparator imbalance. To address this limitation, we analyzed an independent psoriasis skin RNA-seq cohort with bona fide healthy controls (GSE121212), which reproduced the major lesional inflammatory and AMP-associated transcriptomic patterns observed in GSE186063.

Despite these limitations, the consistency of the principal signals across independent cohorts, omics layers, and analytical approaches supports the robustness of the overall systems-level interpretation. In particular, the convergence of gut functional remodeling, immune cell methylome changes, and an AMP-dominant lesional effector program was inferred from within-dataset contrasts and cross-layer directional concordance rather than from direct sample-level pooling or dependence on any single dataset alone. Together, these considerations support the interpretation that the core inflammatory/AMP-dominant lesional signal is robust to comparator context, whereas aggregated lipid/metabolic pathway summaries should be interpreted more conservatively because cohort composition, metadata completeness, and comparator definitions differed across datasets.

## 5. Conclusions

This study presents a multi-omics framework integrating gut microbial functional profiles, systemic immune cell DNA methylation, and lesional skin transcriptomics to delineate convergent molecular remodeling in psoriasis. Across independent public datasets, psoriasis was associated with reduced gut lipid catabolic capacity and lower terminal SCFA-associated functional potential, widespread PBMC and CD8+ T-cell methylome remodeling consistent with systemic immune cell priming, and an AMP-dominant lesional skin program supported by directionality-constrained miRNA–mRNA regulatory links. Together, these findings support a Triple-Hit convergence model in which microbial metabolic remodeling, immune cell epigenetic reprogramming, and cutaneous inflammatory amplification co-occur across compartments in psoriasis.

Although this study was based on inferred functional and cellular estimates from independent public cohorts, the directional consistency of the signals across gut, blood, and skin supports the robustness of this systems-level framework. From a translational perspective, the present model suggests three non-mutually exclusive intervention layers: the restoration of microbial metabolic capacity, particularly SCFA- and lipid-associated functions; attenuation of systemic immune cell epigenetic priming; and suppression of the downstream AMP/IL-36-centered lesional effector program. Rather than prematurely prioritizing a single molecular target, our findings support the view that meaningful therapeutic benefit may ultimately depend on how effectively these upstream, mediator, and effector layers can be decoupled.

Future studies integrating longitudinal sampling, direct metabolite measurements, matched multi-compartment profiling, and mechanistic validation will be necessary to test causality and to determine whether microbial metabolic and immune epigenetic states can be therapeutically modulated alongside existing anti-inflammatory strategies.

## Figures and Tables

**Table 1:** Summary of datasets and analytical outputs.

Layer	Dataset	Omics/Platform	Groups Used (*n*)	Key Outputs
Gut microbiome	GSE239722	Shotgun metagenomics	HC (*n* = 8), PsO-UT ^1^ (*n* = 8)	Taxonomy; functional profiling (KEGG L2/L3, GO)
Systemic (PBMC)	GSE200376	DNA methylation (EPIC 850K)	HC (*n* = 19), PsO-PB ^2^ (*n* = 20)	DMP/DMR Promoter-focused enrichment
Systemic (CD8+ T)	GSE184500	DNA methylation (EPIC 850K)	HC (*n* = 9), PsO-CD8 ^3^ (*n* = 10)	Cell-type EWAS; DMP/DMR
Skin (miRNA)	GSE220586	miRNA array	HC (*n* = 4), PsO-L ^4^ (*n* = 4)	DE-miRNA; target inference (TargetScan/miRDB)
Skin transcriptome	GSE186063	Bulk RNA-seq	AS-HC ^5^ (*n* = 12), PsO-L (*n* = 13)	DEGs; AMP program Immune/stromal deconvolution (MCP-counter)

^1^ PsO-UT (GSE239722): untreated psoriasis samples labeled BT* were used. ^2^ PsO-PB (GSE200376): psoriasis vulgaris peripheral blood samples were used. ^3^ **PsO-CD8 (GSE184500)**: **CD8+ T-cell DNA methylation** profiles from psoriasis patients were used. ^4^ **PsO-L (GSE220586, GSE186063)**: psoriasis **lesional** skin samples were used. ^5^ AS-HC (GSE186063): healthy-appearing skin from patients with ankylosing spondylitis was used as the available within-dataset comparator and not as a bona fide healthy skin control.

## Data Availability

The datasets analyzed in this study are publicly available in the Gene Expression Omnibus (GEO) under accession numbers GSE239722, GSE200376, GSE184500, GSE220586, GSE186063, and GSE121212. The analysis scripts used for data processing and visualization and the derived summary tables/figures are available from the corresponding author upon reasonable request.

## Supplementary Materials

The following supporting information can be downloaded at: https://www.mdpi.com/article/10.3390/life16030516/s1, Figure S1: Taxonomic composition of the gut microbiome in healthy controls and psoriasis patients (GSE239722); Figure S2: Alpha diversity of the gut microbiome at the genus level (GSE239722); Figure S3: Beta diversity of the gut microbiome at the genus level (GSE239722); Figure S4: ssGSEA-based Hallmark pathway activity in lesional skin transcriptomes (GSE186063); Figure S5: Correlation between AMP gene expression and inferred immune/stromal cell population scores in GSE186063 skin transcriptomes; Figure S6: Independent healthy control validation of lesional skin transcriptomic remodeling in psoriasis (GSE121212); Table S1: Cohort demographics and sample characteristics across the public datasets analyzed in main study and external validation; Table S2: Dataset-wise computational workflow used in the Triple-Hit multi-omics framework and external healthy control validation of the skin transcriptomic layer.

## Abbreviations

The following abbreviations are used in this manuscript:

AMP	antimicrobial peptide
AS-HC	healthy-appearing skin from ankylosing spondylitis patients (Control)
BH	Benjamini–Hochberg
CPM	counts per million
DEG	differentially expressed gene
DE-miRNA	differentially expressed microRNA
DMP	differentially methylated position
DMR	differentially methylated region
EPIC	Infinium MethylationEPIC BeadChip (850K)
FDR	false discovery rate
GEO	Gene Expression Omnibus
GO	gene ontology
GSEA	gene set enrichment analysis
HC	healthy control
KEGG	Kyoto Encyclopedia of Genes and Genomes
L2/L3	KEGG Level 2/Level 3 functional hierarchy
logFC	Log2 fold change
NES	normalized enrichment score
ORA	over-representation analysis
PBMC	peripheral blood mononuclear cell
PCoA	principal coordinates analysis
PCA	principal component analysis
PsO	psoriasis
PsO-CD8	CD8+ T cells from psoriasis patients
PsO-L	psoriatic lesional skin
PsO-PB	PBMCs from psoriasis patients
PsO-UT	untreated psoriasis
SCFA	short-chain fatty acid
SRA	Sequence Read Archive
TF	transcription factor
TMM	trimmed mean of M-values

## Appendix A. The Definition of the Gut Lipid Degradation Functional Score (Figure 2d)

To quantify gut microbial lipid degradation-related functional potential (Figure 2d), a composite “Lipid Degradation Functional Score” was computed per sample as the sum of z-scores (standardized across all samples in the cohort) from three KEGG pathway Level 3 categories:

• Fatty acid degradation (ko00071)
• Glycerolipid metabolism (ko00561)
• Glycerophospholipid metabolism (ko00564)

This combination was chosen to represent community-level functional capacity related to lipid catabolism and lipid processing/turnover, enabling an aggregate summary beyond a single pathway.

## Appendix B. Hallmark Gene Set Aggregation Logic (Figure 6d)

To summarize MSigDB Hallmark enrichments into interpretable axes (Figure 6d), the Hallmark gene sets were grouped using a **pre-specified, name-based keyword matchingheuristic** applied to gene set names (case-insensitive; ignore.case = TRUE). This grouping was used **for descriptive axis-level summarization** and was not optimized post hoc to fit the data. The Regular Expressions (Regex) applied in the R script were:

• **Inflammation axis:** grepl (“IL-17|TNF|INTERFERON|NF-KB|INFLAMM”, x, ignore.case = TRUE).

This rule captures the Hallmark gene sets reflecting inflammatory signaling and response programs (e.g., TNFα/NF-κB, interferon responses, and inflammatory response).

• **Lipid metabolism axis:** grepl (“LIPID|FATTY|SPHINGO|CHOLESTEROL”, x, ignore.case = TRUE)

This rule captures lipid-related metabolic programs (e.g., fatty acid metabolism, adipogenesis, and cholesterol homeostasis).

• **Insulin/Metabolic signaling axis:** grepl (“INSULIN|PI3K|AKT|MTOR|GLYCOLYSIS”, x, ignore.case = TRUE). This rule captures anabolic and glycolysis-handling programs spanning PI3K/AKT/mTOR signaling and central carbon metabolism.
