# Supplementary material for: A Triple-Hit Multi-Omics Framework for Psoriasis: Microbial Metabolic Remodeling and Immune Cell Methylome Signature Associated with an AMP-Dominant Lesional Program
Source: Life (Basel). 2026 Mar 20;16(3):516. doi: 10.3390/life16030516 (PMC13027561; doi:10.3390/life16030516)
Supplement: Supplementary file 1 [file life-16-00516-s001.zip › SupplementaryFigure_Revised_v1.pdf]

Supplementary Figure S1. Taxonomic composition of the gut microbiome in healthy controls and psoriasis patients (GSE239722)

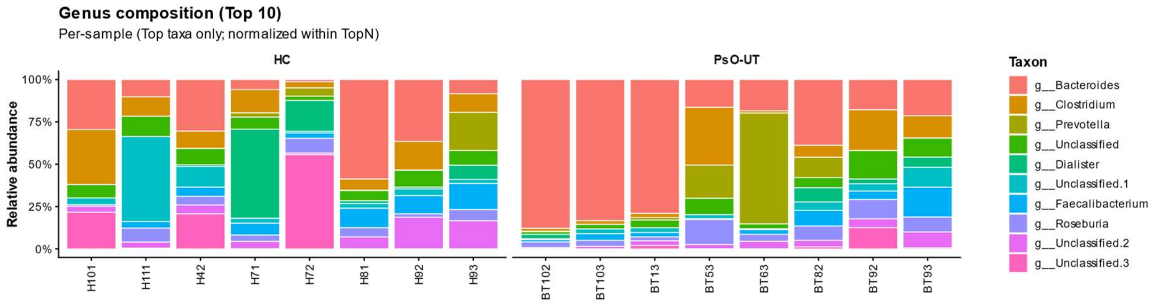

Supplementary Figure S1. Taxonomic composition of the gut microbiome in healthy controls and psoriasis patients.

(S1a) Per-sample stacked bar plots showing the relative abundance of the top 10 most abundant taxa at the **genus level** across healthy controls (HC) and untreated psoriasis samples (PsO-UT), derived from shotgun metagenomic sequencing. For visualization clarity, only the top 10 taxa within each sample are displayed, and relative abundances are normalized within the Top10 taxa. Taxa outside the top 10 are not shown. The plot highlights substantial inter-individual variability in community composition, with psoriasis samples exhibiting heterogeneous dominance patterns, including enrichment of specific genera such as *Bacteroides*, *Prevotella*, and *Clostridium* in distinct subsets of individuals.

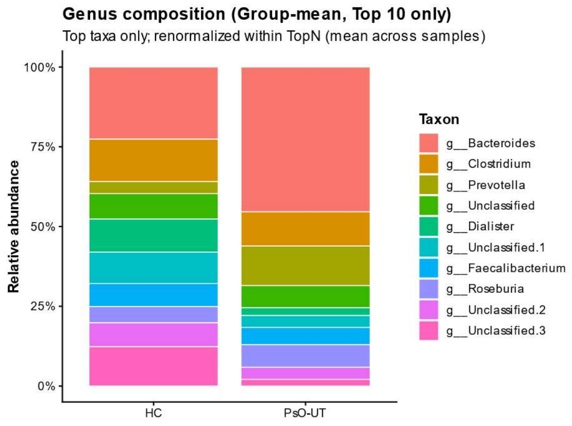

Supplementary Figure S1. Taxonomic composition of the gut microbiome in healthy controls and psoriasis patients.

(S1b) Group-mean stacked bar plots showing the average relative abundance of the top 10 most abundant taxa at the **genus level** in healthy controls (HC) and untreated psoriasis samples (PsO-UT), derived from shotgun metagenomic sequencing. For visualization consistency with the per-sample profiles, only the top 10 taxa are displayed, and relative abundances are normalized within the Top10 taxa based on the mean across samples in each group. This group-level summary illustrates overall shifts in dominant genera between HC and PsO-UT, while masking the substantial inter-individual heterogeneity observed at the per-sample level (S1a), indicating that group-mean profiles primarily reflect broad compositional tendencies rather than uniform disease-specific signatures.

Supplementary Figure S2. Alpha diversity at genus level (GSE239722)

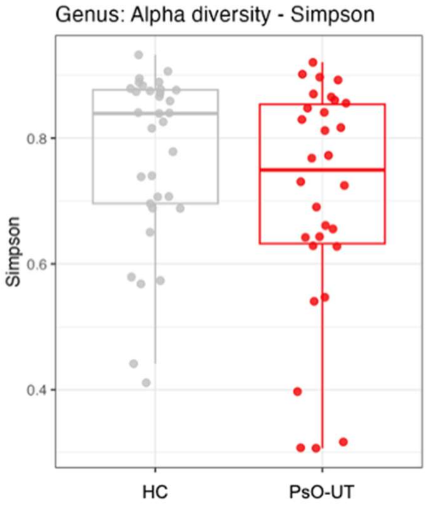

Supplementary Figure S2. Alpha diversity of the gut microbiome at the leading taxonomic level.

(S2) Boxplots showing **genus-level alpha diversity** measured by the **Simpson index** in healthy controls (HC) and untreated psoriasis samples (PsO-UT), based on shotgun metagenomic sequencing. Individual data points represent single samples. While the median Simpson diversity was comparable between groups, psoriasis samples exhibited markedly increased inter-individual variability, with a subset of individuals showing reduced Simpson diversity, indicative of dominance-driven community structures. These results suggest heterogeneous remodeling of gut microbial communities in psoriasis rather than a uniform loss of alpha diversity.

Supplementary Figure S3. Beta diversity at genus level (GSE239722)

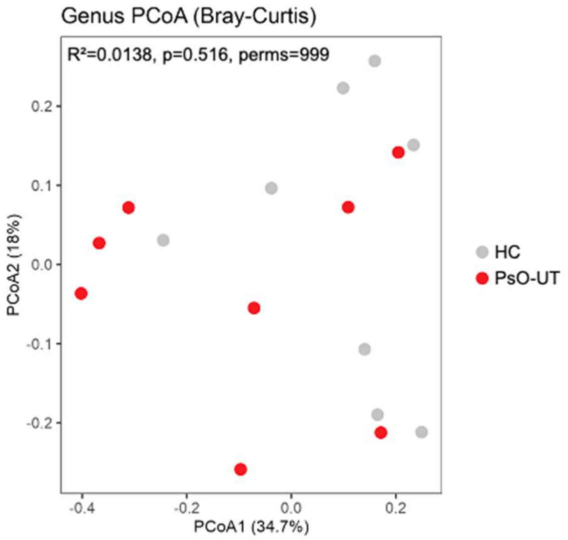

Supplementary Figure S3. Beta diversity of the gut microbiome at the genus level.

(S3) Principal coordinates analysis (PCoA) based on **Bray–Curtis dissimilarity** showing genus-level gut microbiome composition in healthy controls (HC) and untreated psoriasis samples (PsO-UT), derived from shotgun metagenomic sequencing. Group-level differences were assessed using PERMANOVA with 999 permutations, which did not detect a statistically significant separation between groups ($R^2 = 0.0138$, $p = 0.516$). While no clear disease-specific clustering was observed, psoriasis samples displayed a broader dispersion in ordination space, consistent with increased inter-individual heterogeneity rather than a uniform shift in overall community composition.

Supplementary Figure S4. ssGSEA-based Hallmark pathway activity in lesional skin transcriptomes (GSE186063)

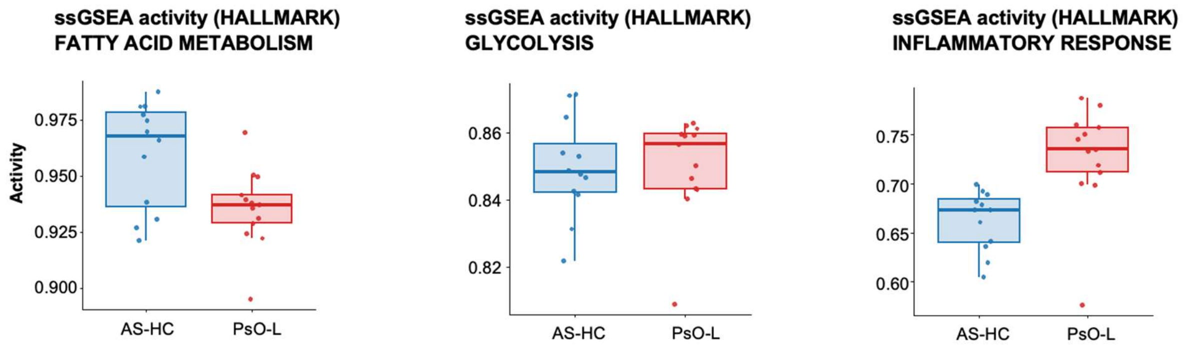

Supplementary Figure S4. ssGSEA-based Hallmark pathway activity in lesional skin transcriptomes.

(S4) Single-sample gene set enrichment analysis (ssGSEA) was applied to bulk RNA-sequencing data from GSE186063 to estimate per-sample activity scores of selected MSigDB Hallmark pathways. Boxplots show ssGSEA activity scores for FATTY ACID METABOLISM, GLYCOLYSIS, and INFLAMMATORY RESPONSE in lesional psoriatic skin (PsO-L) compared with control skin (AS-HC). Each point represents an individual sample. Group-wise distributions illustrate relative pathway activity differences between AS-HC and PsO-L at the single-sample level.

Supplementary Figure S5. Correlation between AMP gene expression and inferred immune/stromal cell population scores in GSE186063 skin transcriptomes

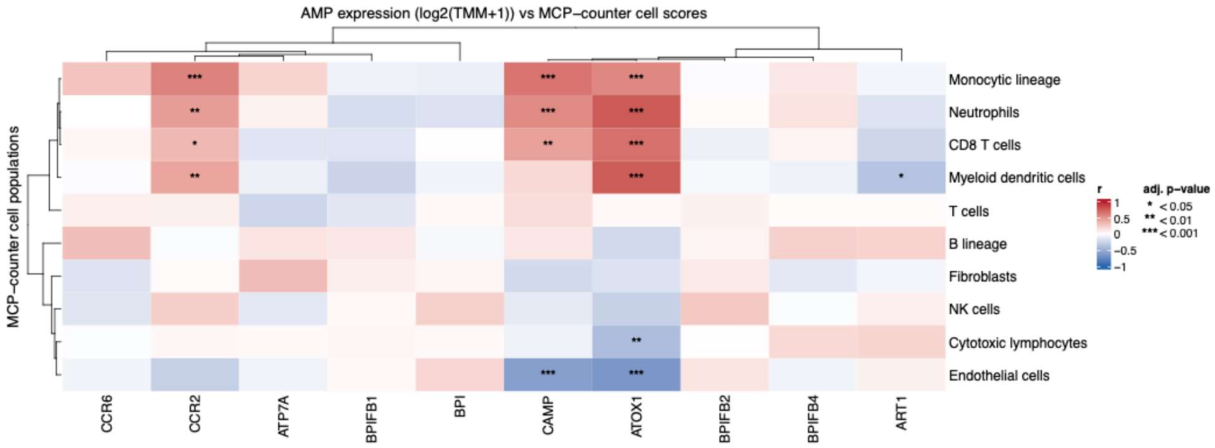

Supplementary Figure S5. Spearman correlation between AMP gene expression and MCP-counter cell/immune population scores in lesional skin transcriptomes.

(S5a) Heatmap displays Spearman's rank correlation coefficients (r) between per-sample expression of antimicrobial peptide (AMP) genes and MCP-counter–estimated cell/immune population scores across GSE186063 skin bulk RNA-seq samples. AMP expression values were computed as log2(TMM-normalized expression + 1), and cell scores were derived using MCP-counter from the same gene-symbol–level expression matrix. For each cell type–gene pair, significance was assessed using correlation test p-values followed by Benjamini–Hochberg FDR correction (adj. p-value). Asterisks indicate FDR thresholds (* < 0.05, ** < 0.01, *** < 0.001). Rows and columns were hierarchically clustered using a distance metric of 1 − Pearson correlation (pairwise complete observations).

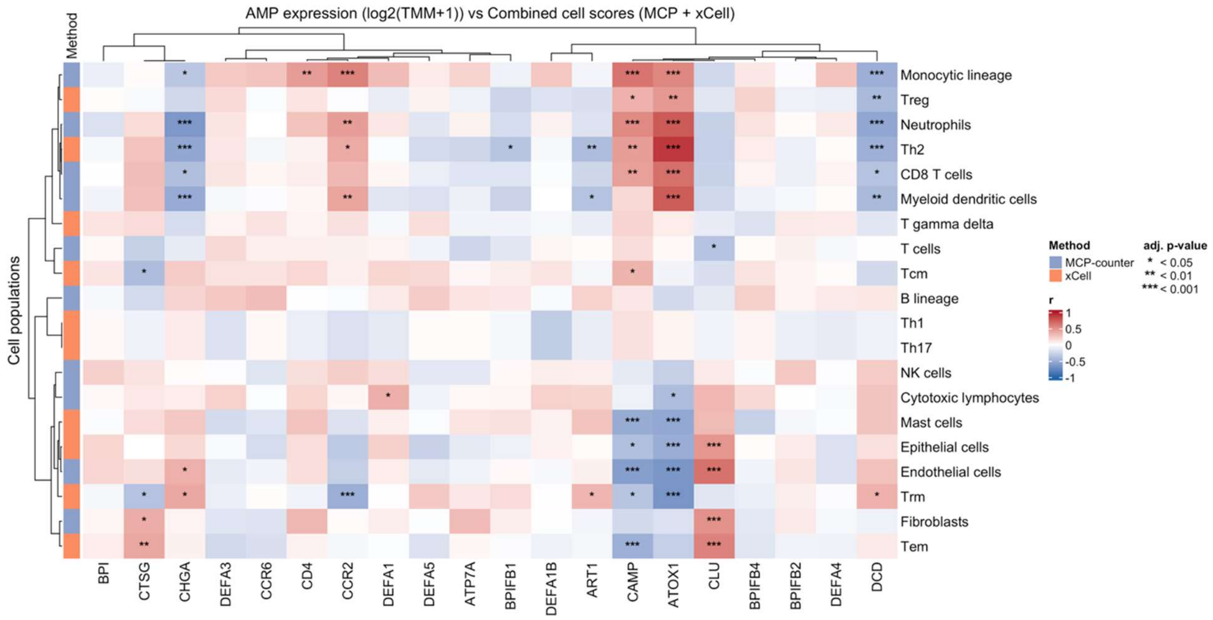

Supplementary Figure S5. Spearman correlation between AMP gene expression and MCP-counter cell/immune population scores in lesional skin transcriptomes.

(S5b) The same analysis repeated using **combined cell population scores derived from MCP-counter and xCell**, with the inference method for each cell population indicated in the left-side annotation. Color represents the correlation coefficient (r; range −1 to 1), and asterisks denote **FDR-adjusted** significance using the same thresholds as in **(S5a).**

Supplementary Figure S6. Independent healthy-control validation of lesional skin transcriptomic remodeling in psoriasis (GSE121212)

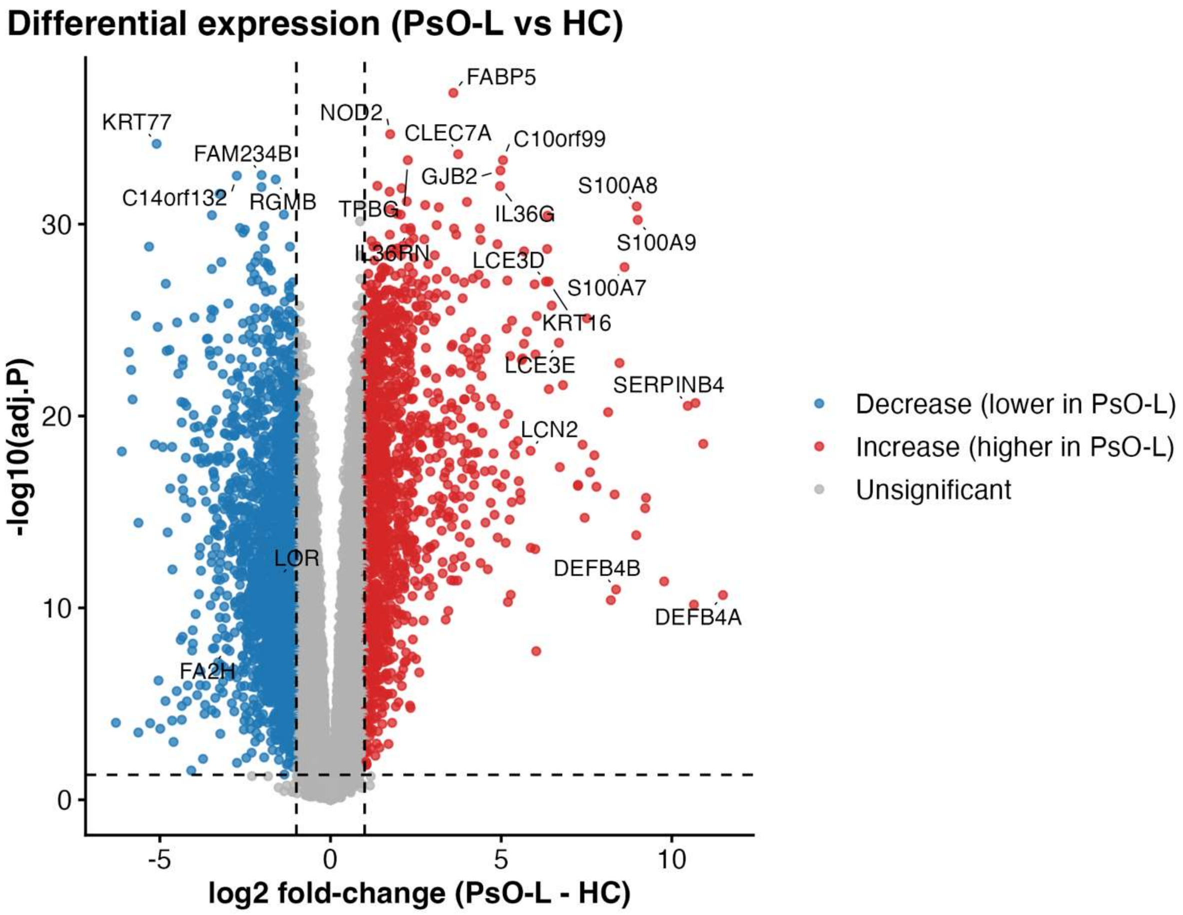

Supplementary Figure S6. Independent healthy-control validation of lesional skin transcriptomic remodeling in psoriasis.

(S6a) Volcano plot showing differential gene expression between lesional psoriatic skin (PsO-L) and healthy control skin (HC) in the GSE121212 bulk RNA-seq dataset. Each point represents one gene. Red and blue points indicate significantly upregulated and downregulated genes, respectively, based on the predefined significance and fold-change thresholds. Representative genes associated with psoriasis lesional inflammation and epidermal stress are annotated.

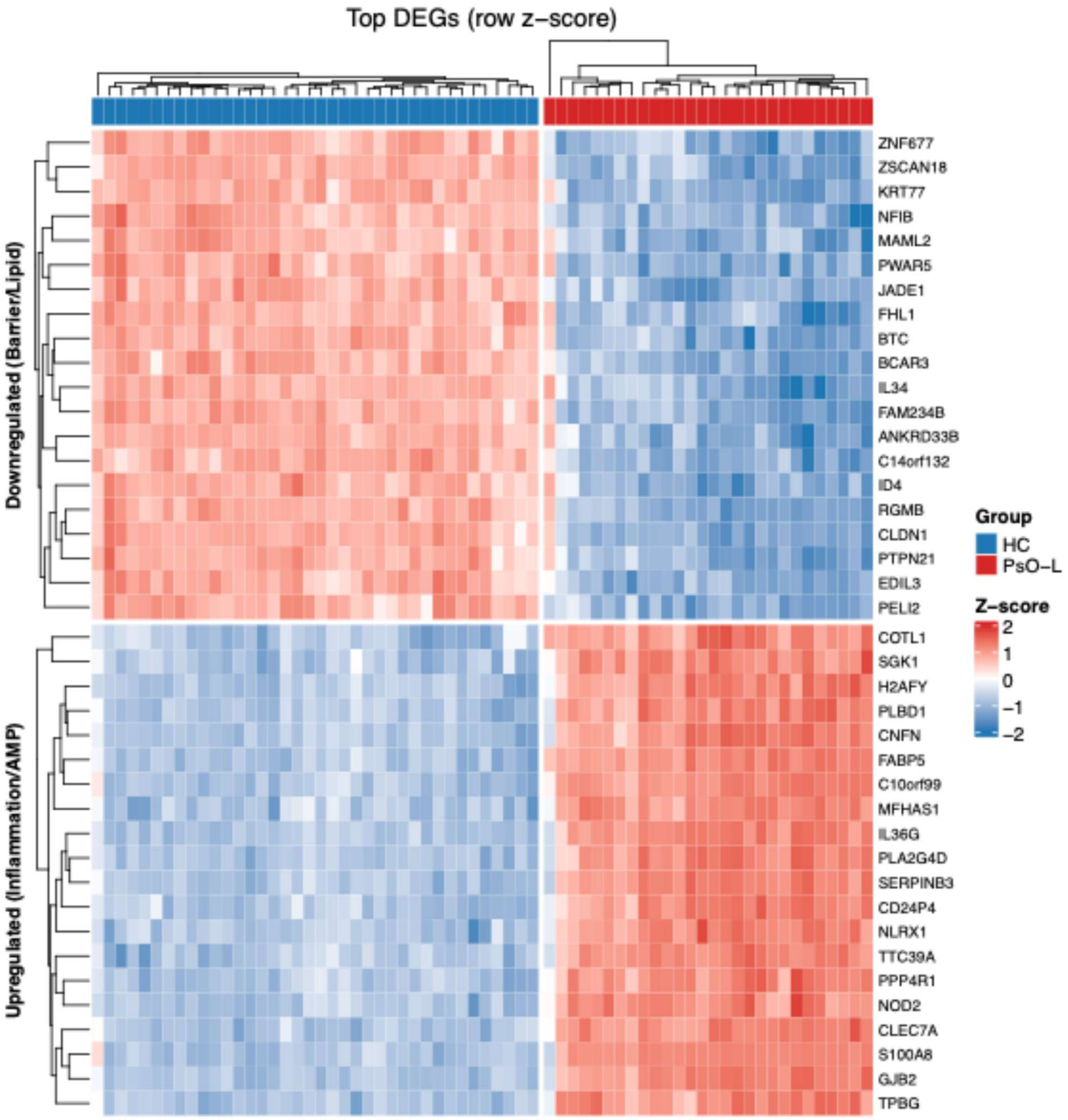

Supplementary Figure S6. Independent healthy-control validation of lesional skin transcriptomic remodeling in psoriasis.

(S6b) Heatmap of representative differentially expressed genes (DEGs) between HC and PsO-L in GSE121212. Expression values are displayed as row z-scores based on normalized expression levels. Samples are grouped by condition, and genes are organized to highlight major expression shifts corresponding to inflammatory/AMP-associated upregulation and barrier/homeostatic downregulation in psoriatic lesions.

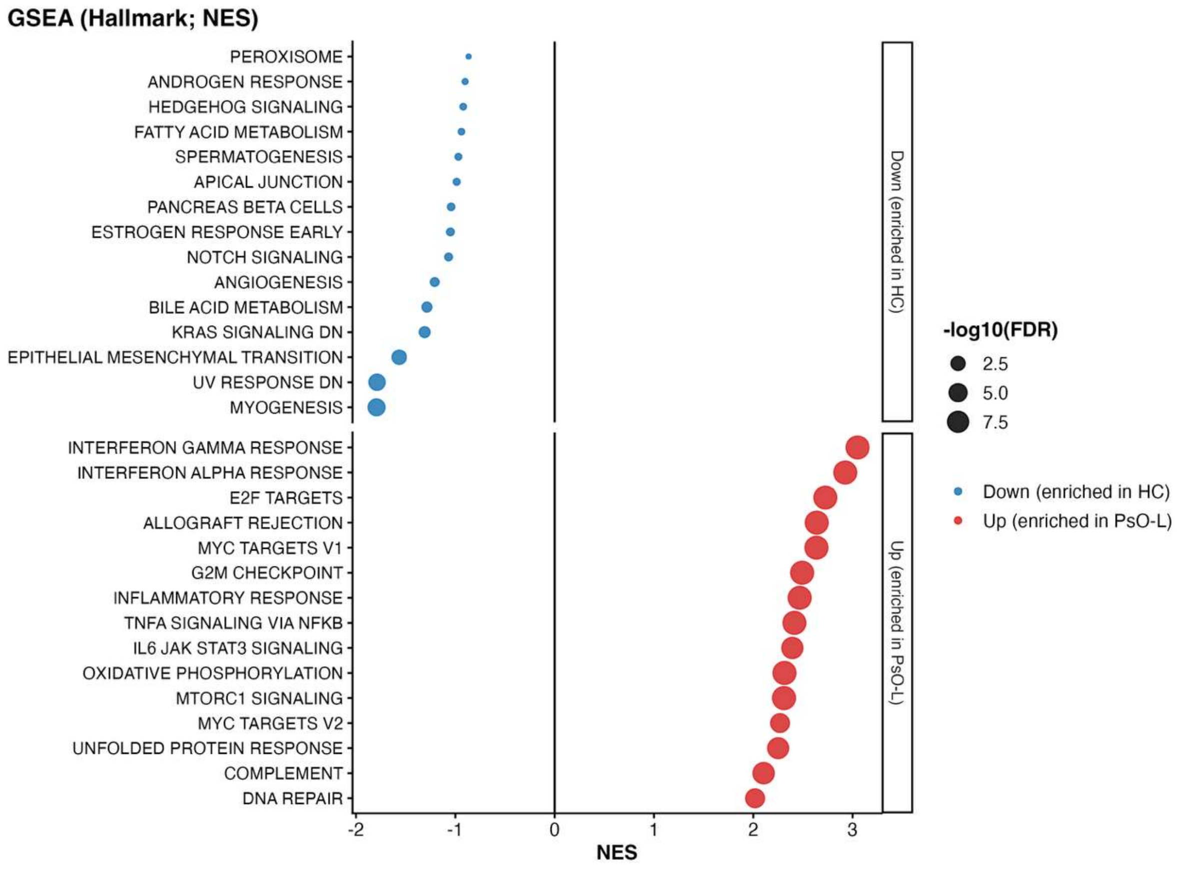

Supplementary Figure S6. Independent healthy-control validation of lesional skin transcriptomic remodeling in psoriasis.

(S6c) Preranked GSEA of MSigDB Hallmark pathways based on the differential expression signature of PsO-L versus HC in GSE121212. Positive normalized enrichment score (NES) values indicate pathways enriched in PsO-L, whereas negative NES values indicate pathways relatively enriched in HC. Dot size represents the significance level as −log10(FDR), and selected leading pathways are shown.

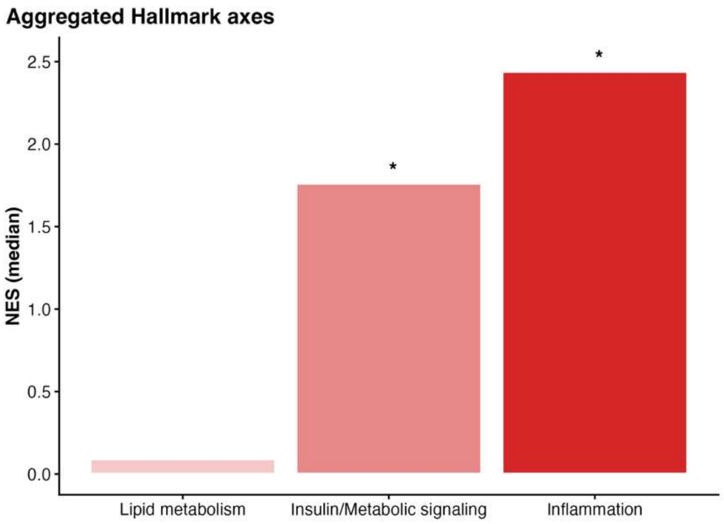

Supplementary Figure S6. Independent healthy-control validation of lesional skin transcriptomic remodeling in psoriasis.

(S6d) Aggregated Hallmark axes summarizing pathway-level remodeling across three conceptual domains in GSE121212: Lipid metabolism, Insulin/Metabolic signaling, and Inflammation. Bars represent the median NES of Hallmark pathways assigned to each axis, providing a compact functional overview of coordinated lesional transcriptomic reprogramming in psoriasis relative to healthy control skin.
