# Supplementary material for: A Triple-Hit Multi-Omics Framework for Psoriasis: Microbial Metabolic Remodeling and Immune Cell Methylome Signature Associated with an AMP-Dominant Lesional Program
Source: Life (Basel). 2026 Mar 20;16(3):516. doi: 10.3390/life16030516 (PMC13027561; doi:10.3390/life16030516)
Supplement: Supplementary file 1 [file life-16-00516-s001.zip › SupplementaryTable_Revised_2nd_v2.pdf]

Supplementary Table S1. Cohort demographics and sample characteristics across the public datasets analyzed in the main study and external validation

Dataset	Group	n	Age	Sex	Annotation
GSE239722 (Gut microbiome)	HC	8	NA	NA	Healthy control
	PsO-UT	8	NA	NA	Plaque psoriasis; untreated
GSE200376 (Systemic, PBMC)	HC	19	48.4 ± 10.9 years; median 51; range 29–65	5F / 14M	Healthy control; PBMC
	PsO-PB	20	49.6 ± 12.5 years; median 50; range 30–68	5F / 15M	Psoriasis vulgaris; PBMC
GSE184500 (Systemic, CD8+ T)	HC	9	32.2 ± 9.2 years; median 27; range 25–51	5F / 4M	Healthy control; purified circulating CD8+ T cells
	PsO-CD8	10	36.8 ± 11.5 years; median 35; range 20–51	3F / 7M	Psoriasis; before treatment; purified circulating CD8+ T cells
GSE220586 (Skin, miRNA)	HC	4	NA	NA	Healthy control; healthy skin
	PsO-L	4	NA	NA	Psoriasis; lesional skin
GSE186063 (Skin, transcriptome)	AS-HC	12	44.2 ± 8.5 years; median 47; range 26–54	3F / 9M	Ankylosing spondylitis; healthy-appearing skin
	PsO-L	13	41.3 ± 13.2 years; median 41; range 20–63	7F / 6M	Psoriasis; lesional skin
GSE121212 (Skin, transcriptome)	HC	38	NA	NA	Healthy control; healthy skin
	PsO-L	28	NA	NA	Psoriasis; lesional skin

Supplementary Table S1. Cohort demographics and sample characteristics across the public datasets analyzed in the main study and external validation.

Values are reported as available from deposited public metadata and/or source-study annotations. Age is presented in years, and sex is shown as female/male counts where available. Annotation consolidates treatment status, lesion status, and other cohort descriptors. NA indicates metadata not available or not uniformly extractable at the group level from deposited public records. Not uniformly available across public cohorts. GSE121212 was used as an external healthy-control validation cohort and was not included in the primary multi-omics integration framework.

Supplementary Table S2. Dataset-wise computational workflow used in the Triple-Hit multi-omics framework and external healthy-control validation of the skin transcriptomic layer

Dataset / layer	Preprocessing / QC	Statistical analysis	Functional / regulatory interpretation	Primary outputs
**GSE239722**	- **Source-study processing**: assembly (MEGAHIT), gene prediction (MetaGeneMark), non-redundant catalog (MMseqs2), KEGG annotation<br>- **Current-study input**: author-provided processed functional profiles<br>- Relative abundance normalization	- Wilcoxon rank-sum test<br>- BH-FDR correction<br>- Targeted comparison of SCFA- and lipid-related pathways	- KEGG functional remodeling<br>- Lipid degradation functional score<br>- Microbial metabolic imbalance summary	- SCFA/lipid pathway reduction<br>- Lipid degradation score<br>- Gut upstream functional remodeling
**GSE200376**	- minfi / Noob normalization<br>- Detection filtering<br>- Remove SNP/cross-reactive probes<br>- Remove sex chromosome probes	- DMP: limma on M-values (Group + Sex + Age)<br>- DMR: DMRcate<br>- Cell composition not regressed out	- Promoter-focused ORA: GO / KEGG / Reactome<br>- Systemic immune epigenetic remodeling	- DMP/DMR landscape<br>- Top DMR signature<br>- Promoter-level pathway enrichment
**GSE184500**	- minfi / Noob normalization<br>- Detection filtering<br>- Remove SNP/cross-reactive probes<br>- Remove sex chromosome probes	- DMR discovery<br>- Cell-type-specific methylation comparison<br>- Psoriasis before-treatment CD8+ layer	- Promoter-focused ORA: GO / KEGG / Reactome<br>- Cytotoxic / trafficking-linked epigenetic signals	- CD8+ DMR landscape<br>- Representative loci<br>- Cell-specific immune epigenetic priming
**GSE220586**	- Quality filtering<br>- Missing-value handling<br>- Low-variance filtering<br>- PCA QC	- Differentially expressed miRNAs<br>- limma-based comparison<br>- FDR-controlled selection	- Target inference databases<br>- Evidence-tier prioritization<br>- Directionality-constrained miRNA–mRNA bridge	- DEM set<br>- High-confidence target network<br>- Regulatory bridge to skin transcriptome

Dataset / layer	Preprocessing / QC	Statistical analysis	Functional / regulatory interpretation	Primary outputs
**GSE186063**	- Sample selection<br>- TMM normalization<br>- logCPM transformation<br>- Gene harmonization	- Differential expression analysis<br>- Volcano / heatmap<br>- Lesion-focused within-dataset comparison	- Hallmark preranked GSEA<br>- 3-axis aggregation<br>- TF activity inference<br>- MCP-counter scoring	- AMP-dominant lesional program<br>- Hallmark inflammatory remodeling<br>- TF / stromal-immune shifts
**GSE121212**	- Count matrix preprocessing<br>- TMM normalization<br>- voom-limma differential expression workflow<br>- HC/PsO-L subset extraction	- Differential expression analysis<br>- Hallmark GSEA<br>- Validation-level comparison	- Inflammatory pathway validation<br>- AMP-associated transcriptomic confirmation<br>- 3-axis Hallmark summary	- Independent validation of major skin-level conclusions derived from GSE186063

Supplementary Table S2. Dataset-wise computational workflow used in the Triple-Hit multi-omics framework and external healthy-control validation of the skin transcriptomic layer.

Each dataset was analyzed independently within its own cohort. Preprocessing was selected according to data modality and analytical input level rather than imposed uniformly across all datasets: Noob normalization was applied to raw Illumina EPIC methylation datasets (GSE200376 and GSE184500), and TMM normalization was applied to count-level bulk RNA-seq datasets (GSE186063 and GSE121212). By contrast, GSE239722 was analyzed using author-provided processed microbial functional abundance profiles, and GSE220586 was analyzed using study-provided normalized miRNA expression values with current-study quality filtering; Noob/TMM was not re-applied to these datasets because such procedures were not methodologically appropriate and could introduce redundant normalization. Cross-layer interpretation was based on directional concordance and biological theme alignment rather than direct sample-level pooling. GSE121212 was included as an external healthy-control validation cohort for the skin transcriptomic layer and was not part of the primary five-dataset Triple-Hit integration framework.
